# Supplementary material for: Use of integrated population models for assessing density-dependence and juvenile survival in Northern Bobwhites (Colinus virginianus)
Source: PeerJ. 2024 Dec 4;12:e18625. doi: 10.7717/peerj.18625 (PMC11624843; doi:10.7717/peerj.18625)
Supplement: Supplemental Information 1 — The lower (2.5%), median (50%), and upper (97.5%) quantiles of posterior samples are shown for each year of the study. D refers to density (birds/ha), and λ refers to population growth rate from year to year+1 for the bobwhite population at the start of April (Apr.Tot superscript) and November (Nov.Tot superscript). ϕ^((J.daily)) is the daily juvenile survival rate, 〖ϕ.μ〗^((B))and ϕ^((B)) are the global mean and yearly (respectively) bi-weekly survival rate during the breeding season (April–September), 〖ϕ.μ〗^((NB))and ϕ^((NB)) are the global mean and yearly (respectively) bi-weekly survival rate during the non-breeding season (October–March), P.μ and P are the global mean and yearly (respectively) per-capita productivity rate, R is the ratio of age and sex classes in November (adult males, adult females, subadults), 〖CS〗^((Act)) is the average covey size, 〖Availability〗^((Act)) is the probability that bobwhite coveys would actively call and be available for detection during November covey count surveys, 〖p.det〗^((Act)) is the probability (conditional on availability) of at least one observer detecting a calling covey during November covey count surveys, 〖γ〗^((B)) is the effect of April density on adult breeding survival, γ^((NB)) is the effect of October density on non-breeding survival, and γ^((prod)) is the effect of monthly density (June–September) on monthly per-capita productivity rates. Subscripts for vital rates correspond to month of the breeding season (m, 1 = June, 2 = July, 3 = August, 4 = September), age during the non-breeding season (a, 1 = adult, 2 = subadult), and sex (s, 1 = male, 2 = female). Estimates are shown for the main model reported in the text using an informative prior on ϕ^((J.daily)) (Informative Prior) and for a model using a vague prior on ϕ^((J.daily)) (Vague Prior). [file peerj-12-18625-s001.docx]

|  |  | Informative Prior | | | Vague Prior | | |
| --- | --- | --- | --- | --- | --- | --- | --- |
| Parameter | Year | Lower | Median | Upper | Lower | Median | Upper |
| $D^{(Apr.Tot)}$ | 1998 | 2.03 | 2.96 | 4.38 | 1.26 | 1.89 | 2.77 |
| $D^{(Apr.Tot)}$ | 1999 | 2.52 | 3.24 | 4.1 | 2.07 | 2.61 | 3.34 |
| $D^{(Apr.Tot)}$ | 2000 | 1.84 | 2.29 | 2.84 | 1.46 | 1.87 | 2.39 |
| $D^{(Apr.Tot)}$ | 2001 | 2.3 | 2.78 | 3.4 | 1.98 | 2.47 | 3.12 |
| $D^{(Apr.Tot)}$ | 2002 | 3.1 | 3.7 | 4.42 | 2.78 | 3.47 | 4.21 |
| $D^{(Apr.Tot)}$ | 2003 | 2.95 | 3.54 | 4.2 | 2.72 | 3.29 | 3.96 |
| $D^{(Apr.Tot)}$ | 2004 | 3.28 | 3.93 | 4.67 | 3.04 | 3.7 | 4.43 |
| $D^{(Apr.Tot)}$ | 2005 | 2.62 | 3.18 | 3.74 | 2.31 | 2.85 | 3.45 |
| $D^{(Apr.Tot)}$ | 2006 | 2.33 | 2.86 | 3.45 | 2.13 | 2.67 | 3.33 |
| $D^{(Apr.Tot)}$ | 2007 | 1.72 | 2.13 | 2.64 | 1.51 | 1.9 | 2.36 |
| $D^{(Apr.Tot)}$ | 2008 | 2.05 | 2.52 | 3.1 | 1.84 | 2.37 | 3.06 |
| $D^{(Apr.Tot)}$ | 2009 | 2.52 | 3.09 | 3.78 | 2.46 | 3.18 | 4.12 |
| $D^{(Apr.Tot)}$ | 2010 | 2.74 | 3.29 | 3.99 | 2.58 | 3.28 | 4.11 |
| $D^{(Apr.Tot)}$ | 2011 | 2.32 | 2.83 | 3.47 | 2.21 | 2.77 | 3.56 |
| $D^{(Apr.Tot)}$ | 2012 | 2.24 | 2.72 | 3.24 | 1.87 | 2.32 | 2.83 |
| $D^{(Apr.Tot)}$ | 2013 | 3.43 | 4.07 | 4.83 | 3.04 | 3.74 | 4.45 |
| $D^{(Apr.Tot)}$ | 2014 | 3.58 | 4.29 | 5.09 | 3.41 | 4.22 | 5.21 |
| $D^{(Apr.Tot)}$ | 2015 | 2.41 | 2.9 | 3.5 | 2.09 | 2.57 | 3.14 |
| $D^{(Apr.Tot)}$ | 2016 | 2.99 | 3.57 | 4.29 | 2.8 | 3.38 | 4.09 |
| $D^{(Apr.Tot)}$ | 2017 | 3.33 | 4 | 4.85 | 3.32 | 4.09 | 4.95 |
| $D^{(Apr.Tot)}$ | 2018 | 3.37 | 4.05 | 4.87 | 3.35 | 4.16 | 5.27 |
| $D^{(Apr.Tot)}$ | 2019 | 3.14 | 3.78 | 4.55 | 2.88 | 3.52 | 4.51 |
| $D^{(Apr.Tot)}$ | 2020 | 3.82 | 4.61 | 5.41 | 3.77 | 4.52 | 5.57 |
| $D^{(Apr.Tot)}$ | 2021 | 3.77 | 4.47 | 5.25 | 3.65 | 4.28 | 5.05 |
| $D^{(Apr.Tot)}$ | 2022 | 3.78 | 4.4 | 5.16 | 3.88 | 4.5 | 5.28 |
| $\lambda^{(Apr.Tot)}$ | 1998 | 0.8 | 1.1 | 1.45 | 0.98 | 1.39 | 1.99 |
| $\lambda^{(Apr.Tot)}$ | 1999 | 0.56 | 0.71 | 0.89 | 0.53 | 0.71 | 0.93 |
| $\lambda^{(Apr.Tot)}$ | 2000 | 0.97 | 1.22 | 1.51 | 1.01 | 1.32 | 1.72 |
| $\lambda^{(Apr.Tot)}$ | 2001 | 1.11 | 1.33 | 1.58 | 1.15 | 1.4 | 1.72 |
| $\lambda^{(Apr.Tot)}$ | 2002 | 0.81 | 0.96 | 1.12 | 0.8 | 0.96 | 1.14 |
| $\lambda^{(Apr.Tot)}$ | 2003 | 0.93 | 1.11 | 1.32 | 0.89 | 1.12 | 1.41 |
| $\lambda^{(Apr.Tot)}$ | 2004 | 0.67 | 0.81 | 0.95 | 0.63 | 0.77 | 0.93 |
| $\lambda^{(Apr.Tot)}$ | 2005 | 0.74 | 0.9 | 1.07 | 0.76 | 0.94 | 1.15 |
| $\lambda^{(Apr.Tot)}$ | 2006 | 0.62 | 0.75 | 0.89 | 0.57 | 0.71 | 0.88 |
| $\lambda^{(Apr.Tot)}$ | 2007 | 0.98 | 1.18 | 1.44 | 0.96 | 1.26 | 1.61 |
| $\lambda^{(Apr.Tot)}$ | 2008 | 0.998 | 1.23 | 1.5 | 1.03 | 1.34 | 1.75 |
| $\lambda^{(Apr.Tot)}$ | 2009 | 0.86 | 1.07 | 1.31 | 0.79 | 1.03 | 1.35 |
| $\lambda^{(Apr.Tot)}$ | 2010 | 0.71 | 0.86 | 1.04 | 0.63 | 0.85 | 1.14 |
| $\lambda^{(Apr.Tot)}$ | 2011 | 0.78 | 0.96 | 1.18 | 0.62 | 0.84 | 1.09 |
| $\lambda^{(Apr.Tot)}$ | 2012 | 1.24 | 1.5 | 1.83 | 1.25 | 1.61 | 2.05 |
| $\lambda^{(Apr.Tot)}$ | 2013 | 0.88 | 1.05 | 1.27 | 0.89 | 1.14 | 1.44 |
| $\lambda^{(Apr.Tot)}$ | 2014 | 0.55 | 0.68 | 0.83 | 0.47 | 0.61 | 0.78 |
| $\lambda^{(Apr.Tot)}$ | 2015 | 1.04 | 1.23 | 1.46 | 1.07 | 1.32 | 1.62 |
| $\lambda^{(Apr.Tot)}$ | 2016 | 0.93 | 1.12 | 1.36 | 0.98 | 1.2 | 1.51 |
| $\lambda^{(Apr.Tot)}$ | 2017 | 0.85 | 1.02 | 1.22 | 0.81 | 1.02 | 1.3 |
| $\lambda^{(Apr.Tot)}$ | 2018 | 0.78 | 0.93 | 1.1 | 0.68 | 0.85 | 1.05 |
| $\lambda^{(Apr.Tot)}$ | 2019 | 1.03 | 1.21 | 1.43 | 1.02 | 1.29 | 1.57 |
| $\lambda^{(Apr.Tot)}$ | 2020 | 0.82 | 0.97 | 1.13 | 0.78 | 0.94 | 1.14 |
| $\lambda^{(Apr.Tot)}$ | 2021 | 0.84 | 0.99 | 1.17 | 0.87 | 1.05 | 1.26 |
| $D^{(Apr.Tot)}$ | 1998 | 3.74 | 4.8 | 6.07 | 3.17 | 4.05 | 5.19 |
| $D^{(Apr.Tot)}$ | 1999 | 3.53 | 4.4 | 5.5 | 3.25 | 4.03 | 5.01 |
| $D^{(Apr.Tot)}$ | 2000 | 3.56 | 4.33 | 5.28 | 3.34 | 4.13 | 5.22 |
| $D^{(Apr.Tot)}$ | 2001 | 4.64 | 5.54 | 6.62 | 4.37 | 5.44 | 6.62 |
| $D^{(Apr.Tot)}$ | 2002 | 4.48 | 5.35 | 6.32 | 4.33 | 5.24 | 6.29 |
| $D^{(Apr.Tot)}$ | 2003 | 4.83 | 5.79 | 6.86 | 4.74 | 5.71 | 6.8 |
| $D^{(Apr.Tot)}$ | 2004 | 4.14 | 4.97 | 5.85 | 3.98 | 4.83 | 5.82 |
| $D^{(Apr.Tot)}$ | 2005 | 3.62 | 4.41 | 5.23 | 3.56 | 4.42 | 5.44 |
| $D^{(Apr.Tot)}$ | 2006 | 2.67 | 3.28 | 4.02 | 2.58 | 3.2 | 3.98 |
| $D^{(Apr.Tot)}$ | 2007 | 2.95 | 3.63 | 4.46 | 2.92 | 3.69 | 4.74 |
| $D^{(Apr.Tot)}$ | 2008 | 3.88 | 4.7 | 5.66 | 4.21 | 5.31 | 6.65 |
| $D^{(Apr.Tot)}$ | 2009 | 4.18 | 5 | 6 | 4.23 | 5.31 | 6.58 |
| $D^{(Apr.Tot)}$ | 2010 | 3.47 | 4.19 | 5.13 | 3.5 | 4.35 | 5.58 |
| $D^{(Apr.Tot)}$ | 2011 | 3.36 | 4.05 | 4.85 | 3.04 | 3.76 | 4.55 |
| $D^{(Apr.Tot)}$ | 2012 | 4.58 | 5.39 | 6.36 | 4.29 | 5.23 | 6.25 |
| $D^{(Apr.Tot)}$ | 2013 | 5.28 | 6.28 | 7.39 | 5.46 | 6.65 | 8.13 |
| $D^{(Apr.Tot)}$ | 2014 | 3.55 | 4.28 | 5.12 | 3.46 | 4.21 | 5.16 |
| $D^{(Apr.Tot)}$ | 2015 | 4.09 | 4.85 | 5.79 | 4.09 | 4.87 | 5.85 |
| $D^{(Apr.Tot)}$ | 2016 | 4.57 | 5.42 | 6.59 | 4.86 | 5.96 | 7.1 |
| $D^{(Apr.Tot)}$ | 2017 | 4.65 | 5.58 | 6.72 | 4.99 | 6.15 | 7.84 |
| $D^{(Apr.Tot)}$ | 2018 | 4.6 | 5.52 | 6.64 | 4.66 | 5.67 | 7.2 |
| $D^{(Apr.Tot)}$ | 2019 | 5.3 | 6.36 | 7.46 | 5.53 | 6.64 | 8.12 |
| $D^{(Apr.Tot)}$ | 2020 | 5.09 | 6.05 | 7.09 | 5.28 | 6.13 | 7.23 |
| $D^{(Apr.Tot)}$ | 2021 | 5.91 | 6.83 | 7.91 | 6.64 | 7.64 | 8.83 |
| $D^{(Apr.Tot)}$ | 2022 | 4.62 | 5.41 | 6.29 | 5.49 | 6.46 | 7.57 |
| $\lambda^{(Apr.Tot)}$ | 1998 | 0.76 | 0.92 | 1.1 | 0.79 | 1 | 1.24 |
| $\lambda^{(Apr.Tot)}$ | 1999 | 0.79 | 0.98 | 1.24 | 0.79 | 1.02 | 1.33 |
| $\lambda^{(Apr.Tot)}$ | 2000 | 1.06 | 1.28 | 1.53 | 1.07 | 1.31 | 1.63 |
| $\lambda^{(Apr.Tot)}$ | 2001 | 0.82 | 0.96 | 1.12 | 0.81 | 0.97 | 1.14 |
| $\lambda^{(Apr.Tot)}$ | 2002 | 0.91 | 1.08 | 1.27 | 0.87 | 1.09 | 1.36 |
| $\lambda^{(Apr.Tot)}$ | 2003 | 0.72 | 0.86 | 1.01 | 0.7 | 0.85 | 1.02 |
| $\lambda^{(Apr.Tot)}$ | 2004 | 0.74 | 0.89 | 1.06 | 0.74 | 0.91 | 1.11 |
| $\lambda^{(Apr.Tot)}$ | 2005 | 0.63 | 0.75 | 0.89 | 0.59 | 0.72 | 0.89 |
| $\lambda^{(Apr.Tot)}$ | 2006 | 0.91 | 1.11 | 1.33 | 0.9 | 1.16 | 1.48 |
| $\lambda^{(Apr.Tot)}$ | 2007 | 1.07 | 1.29 | 1.56 | 1.13 | 1.43 | 1.84 |
| $\lambda^{(Apr.Tot)}$ | 2008 | 0.87 | 1.07 | 1.29 | 0.78 | 1 | 1.3 |
| $\lambda^{(Apr.Tot)}$ | 2009 | 0.69 | 0.84 | 1.03 | 0.62 | 0.82 | 1.1 |
| $\lambda^{(Apr.Tot)}$ | 2010 | 0.79 | 0.97 | 1.17 | 0.64 | 0.87 | 1.12 |
| $\lambda^{(Apr.Tot)}$ | 2011 | 1.1 | 1.33 | 1.62 | 1.09 | 1.4 | 1.77 |
| $\lambda^{(Apr.Tot)}$ | 2012 | 0.99 | 1.16 | 1.38 | 1.01 | 1.28 | 1.6 |
| $\lambda^{(Apr.Tot)}$ | 2013 | 0.56 | 0.68 | 0.82 | 0.5 | 0.63 | 0.8 |
| $\lambda^{(Apr.Tot)}$ | 2014 | 0.96 | 1.14 | 1.34 | 0.94 | 1.16 | 1.42 |
| $\lambda^{(Apr.Tot)}$ | 2015 | 0.94 | 1.12 | 1.35 | 0.998 | 1.21 | 1.51 |
| $\lambda^{(Apr.Tot)}$ | 2016 | 0.85 | 1.03 | 1.23 | 0.84 | 1.04 | 1.31 |
| $\lambda^{(Apr.Tot)}$ | 2017 | 0.84 | 0.99 | 1.17 | 0.75 | 0.92 | 1.13 |
| $\lambda^{(Apr.Tot)}$ | 2018 | 0.98 | 1.15 | 1.35 | 0.93 | 1.17 | 1.44 |
| $\lambda^{(Apr.Tot)}$ | 2019 | 0.81 | 0.95 | 1.1 | 0.77 | 0.92 | 1.11 |
| $\lambda^{(Apr.Tot)}$ | 2020 | 0.98 | 1.13 | 1.31 | 1.06 | 1.25 | 1.47 |
| $\lambda^{(Apr.Tot)}$ | 2021 | 0.68 | 0.79 | 0.92 | 0.71 | 0.85 | 1.001 |
| $\phi_{m=1}^{\left( J.daily \right)}$ | 1998 | 0.986 | 0.989 | 0.991 | 0.988 | 0.992 | 0.995 |
| $\phi_{m=1}^{\left( J.daily \right)}$ | 1999 | 0.988 | 0.99 | 0.992 | 0.99 | 0.993 | 0.996 |
| $\phi_{m=1}^{\left( J.daily \right)}$ | 2000 | 0.99 | 0.992 | 0.994 | 0.994 | 0.998 | 1 |
| $\phi_{m=1}^{\left( J.daily \right)}$ | 2001 | 0.989 | 0.991 | 0.993 | 0.99 | 0.993 | 0.996 |
| $\phi_{m=1}^{\left( J.daily \right)}$ | 2002 | 0.988 | 0.99 | 0.992 | 0.989 | 0.991 | 0.994 |
| $\phi_{m=1}^{\left( J.daily \right)}$ | 2003 | 0.989 | 0.991 | 0.993 | 0.991 | 0.994 | 0.997 |
| $\phi_{m=1}^{\left( J.daily \right)}$ | 2004 | 0.986 | 0.988 | 0.99 | 0.987 | 0.989 | 0.992 |
| $\phi_{m=1}^{\left( J.daily \right)}$ | 2005 | 0.989 | 0.991 | 0.993 | 0.993 | 0.997 | 0.999 |
| $\phi_{m=1}^{\left( J.daily \right)}$ | 2006 | 0.989 | 0.991 | 0.993 | 0.991 | 0.994 | 0.997 |
| $\phi_{m=1}^{\left( J.daily \right)}$ | 2007 | 0.989 | 0.992 | 0.994 | 0.992 | 0.996 | 0.999 |
| $\phi_{m=1}^{\left( J.daily \right)}$ | 2008 | 0.989 | 0.992 | 0.993 | 0.993 | 0.996 | 0.999 |
| $\phi_{m=1}^{\left( J.daily \right)}$ | 2009 | 0.989 | 0.991 | 0.993 | 0.991 | 0.995 | 0.999 |
| $\phi_{m=1}^{\left( J.daily \right)}$ | 2010 | 0.99 | 0.992 | 0.994 | 0.991 | 0.995 | 0.998 |
| $\phi_{m=1}^{\left( J.daily \right)}$ | 2011 | 0.988 | 0.991 | 0.993 | 0.99 | 0.995 | 0.999 |
| $\phi_{m=1}^{\left( J.daily \right)}$ | 2012 | 0.99 | 0.992 | 0.994 | 0.993 | 0.997 | 0.999 |
| $\phi_{m=1}^{\left( J.daily \right)}$ | 2013 | 0.989 | 0.991 | 0.993 | 0.992 | 0.995 | 0.998 |
| $\phi_{m=1}^{\left( J.daily \right)}$ | 2014 | 0.991 | 0.993 | 0.995 | 0.997 | 0.999 | 1 |
| $\phi_{m=1}^{\left( J.daily \right)}$ | 2015 | 0.99 | 0.992 | 0.994 | 0.995 | 0.998 | 1 |
| $\phi_{m=1}^{\left( J.daily \right)}$ | 2016 | 0.99 | 0.992 | 0.994 | 0.995 | 0.998 | 1 |
| $\phi_{m=1}^{\left( J.daily \right)}$ | 2017 | 0.988 | 0.991 | 0.993 | 0.99 | 0.994 | 0.998 |
| $\phi_{m=1}^{\left( J.daily \right)}$ | 2018 | 0.99 | 0.993 | 0.994 | 0.996 | 0.999 | 1 |
| $\phi_{m=1}^{\left( J.daily \right)}$ | 2019 | 0.99 | 0.992 | 0.994 | 0.994 | 0.998 | 1 |
| $\phi_{m=1}^{\left( J.daily \right)}$ | 2020 | 0.993 | 0.995 | 0.996 | 0.997 | 0.999 | 1 |
| $\phi_{m=1}^{\left( J.daily \right)}$ | 2021 | 0.992 | 0.994 | 0.995 | 0.997 | 0.999 | 1 |
| $\phi_{m=1}^{\left( J.daily \right)}$ | 2022 | 0.992 | 0.994 | 0.995 | 0.997 | 0.999 | 1 |
| $\phi_{m=2}^{\left( J.daily \right)}$ | 1998 | 0.99 | 0.993 | 0.995 | 0.995 | 0.998 | 1 |
| $\phi_{m=2}^{\left( J.daily \right)}$ | 1999 | 0.989 | 0.992 | 0.994 | 0.991 | 0.995 | 0.998 |
| $\phi_{m=2}^{\left( J.daily \right)}$ | 2000 | 0.985 | 0.989 | 0.992 | 0.984 | 0.989 | 0.994 |
| $\phi_{m=2}^{\left( J.daily \right)}$ | 2001 | 0.993 | 0.995 | 0.996 | 0.998 | 0.999 | 1 |
| $\phi_{m=2}^{\left( J.daily \right)}$ | 2002 | 0.992 | 0.994 | 0.995 | 0.996 | 0.999 | 1 |
| $\phi_{m=2}^{\left( J.daily \right)}$ | 2003 | 0.989 | 0.992 | 0.994 | 0.989 | 0.993 | 0.997 |
| $\phi_{m=2}^{\left( J.daily \right)}$ | 2004 | 0.993 | 0.994 | 0.996 | 0.997 | 0.999 | 1 |
| $\phi_{m=2}^{\left( J.daily \right)}$ | 2005 | 0.99 | 0.992 | 0.995 | 0.993 | 0.997 | 1 |
| $\phi_{m=2}^{\left( J.daily \right)}$ | 2006 | 0.991 | 0.993 | 0.995 | 0.996 | 0.999 | 1 |
| $\phi_{m=2}^{\left( J.daily \right)}$ | 2007 | 0.99 | 0.993 | 0.995 | 0.995 | 0.999 | 1 |
| $\phi_{m=2}^{\left( J.daily \right)}$ | 2008 | 0.991 | 0.993 | 0.995 | 0.994 | 0.998 | 1 |
| $\phi_{m=2}^{\left( J.daily \right)}$ | 2009 | 0.986 | 0.99 | 0.993 | 0.98 | 0.988 | 0.993 |
| $\phi_{m=2}^{\left( J.daily \right)}$ | 2010 | 0.987 | 0.99 | 0.993 | 0.984 | 0.99 | 0.996 |
| $\phi_{m=2}^{\left( J.daily \right)}$ | 2011 | 0.989 | 0.992 | 0.994 | 0.987 | 0.993 | 0.999 |
| $\phi_{m=2}^{\left( J.daily \right)}$ | 2012 | 0.99 | 0.993 | 0.995 | 0.994 | 0.998 | 1 |
| $\phi_{m=2}^{\left( J.daily \right)}$ | 2013 | 0.988 | 0.992 | 0.994 | 0.989 | 0.995 | 0.999 |
| $\phi_{m=2}^{\left( J.daily \right)}$ | 2014 | 0.989 | 0.992 | 0.995 | 0.991 | 0.997 | 1 |
| $\phi_{m=2}^{\left( J.daily \right)}$ | 2015 | 0.988 | 0.992 | 0.994 | 0.99 | 0.997 | 1 |
| $\phi_{m=2}^{\left( J.daily \right)}$ | 2016 | 0.99 | 0.993 | 0.995 | 0.995 | 0.999 | 1 |
| $\phi_{m=2}^{\left( J.daily \right)}$ | 2017 | 0.99 | 0.993 | 0.995 | 0.992 | 0.997 | 1 |
| $\phi_{m=2}^{\left( J.daily \right)}$ | 2018 | 0.989 | 0.991 | 0.994 | 0.987 | 0.992 | 0.997 |
| $\phi_{m=2}^{\left( J.daily \right)}$ | 2019 | 0.99 | 0.993 | 0.995 | 0.993 | 0.997 | 1 |
| $\phi_{m=2}^{\left( J.daily \right)}$ | 2020 | 0.992 | 0.994 | 0.996 | 0.997 | 0.999 | 1 |
| $\phi_{m=2}^{\left( J.daily \right)}$ | 2021 | 0.991 | 0.993 | 0.995 | 0.995 | 0.998 | 1 |
| $\phi_{m=2}^{\left( J.daily \right)}$ | 2022 | 0.988 | 0.991 | 0.994 | 0.992 | 0.997 | 1 |
| $\phi_{m=3}^{\left( J.daily \right)}$ | 1998 | 0.985 | 0.99 | 0.993 | 0.987 | 0.995 | 0.999 |
| $\phi_{m=3}^{\left( J.daily \right)}$ | 1999 | 0.988 | 0.991 | 0.994 | 0.992 | 0.997 | 1 |
| $\phi_{m=3}^{\left( J.daily \right)}$ | 2000 | 0.986 | 0.99 | 0.993 | 0.986 | 0.995 | 0.999 |
| $\phi_{m=3}^{\left( J.daily \right)}$ | 2001 | 0.987 | 0.991 | 0.993 | 0.988 | 0.994 | 0.999 |
| $\phi_{m=3}^{\left( J.daily \right)}$ | 2002 | 0.985 | 0.99 | 0.993 | 0.985 | 0.992 | 0.998 |
| $\phi_{m=3}^{\left( J.daily \right)}$ | 2003 | 0.987 | 0.991 | 0.994 | 0.988 | 0.995 | 0.999 |
| $\phi_{m=3}^{\left( J.daily \right)}$ | 2004 | 0.985 | 0.989 | 0.992 | 0.982 | 0.99 | 0.998 |
| $\phi_{m=3}^{\left( J.daily \right)}$ | 2005 | 0.989 | 0.992 | 0.994 | 0.994 | 0.998 | 1 |
| $\phi_{m=3}^{\left( J.daily \right)}$ | 2006 | 0.988 | 0.991 | 0.994 | 0.991 | 0.997 | 1 |
| $\phi_{m=3}^{\left( J.daily \right)}$ | 2007 | 0.986 | 0.99 | 0.993 | 0.986 | 0.994 | 0.999 |
| $\phi_{m=3}^{\left( J.daily \right)}$ | 2008 | 0.989 | 0.992 | 0.994 | 0.994 | 0.998 | 1 |
| $\phi_{m=3}^{\left( J.daily \right)}$ | 2009 | 0.989 | 0.992 | 0.994 | 0.994 | 0.998 | 1 |
| $\phi_{m=3}^{\left( J.daily \right)}$ | 2010 | 0.984 | 0.989 | 0.992 | 0.977 | 0.987 | 0.997 |
| $\phi_{m=3}^{\left( J.daily \right)}$ | 2011 | 0.989 | 0.992 | 0.995 | 0.995 | 0.999 | 1 |
| $\phi_{m=3}^{\left( J.daily \right)}$ | 2012 | 0.986 | 0.99 | 0.993 | 0.984 | 0.993 | 0.999 |
| $\phi_{m=3}^{\left( J.daily \right)}$ | 2013 | 0.988 | 0.991 | 0.994 | 0.993 | 0.998 | 1 |
| $\phi_{m=3}^{\left( J.daily \right)}$ | 2014 | 0.984 | 0.989 | 0.993 | 0.974 | 0.987 | 0.998 |
| $\phi_{m=3}^{\left( J.daily \right)}$ | 2015 | 0.986 | 0.99 | 0.993 | 0.987 | 0.996 | 0.999 |
| $\phi_{m=3}^{\left( J.daily \right)}$ | 2016 | 0.987 | 0.991 | 0.994 | 0.991 | 0.997 | 1 |
| $\phi_{m=3}^{\left( J.daily \right)}$ | 2017 | 0.989 | 0.992 | 0.995 | 0.995 | 0.999 | 1 |
| $\phi_{m=3}^{\left( J.daily \right)}$ | 2018 | 0.988 | 0.991 | 0.994 | 0.99 | 0.997 | 1 |
| $\phi_{m=3}^{\left( J.daily \right)}$ | 2019 | 0.984 | 0.989 | 0.992 | 0.982 | 0.991 | 0.998 |
| $\phi_{m=3}^{\left( J.daily \right)}$ | 2020 | 0.976 | 0.983 | 0.988 | 0.97 | 0.978 | 0.985 |
| $\phi_{m=3}^{\left( J.daily \right)}$ | 2021 | 0.987 | 0.991 | 0.994 | 0.991 | 0.997 | 0.999 |
| $\phi_{m=3}^{\left( J.daily \right)}$ | 2022 | 0.986 | 0.99 | 0.993 | 0.99 | 0.997 | 1 |
| $\phi_{m=4}^{\left( J.daily \right)}$ | 1998 | 0.989 | 0.993 | 0.995 | 0.977 | 0.994 | 0.999 |
| $\phi_{m=4}^{\left( J.daily \right)}$ | 1999 | 0.989 | 0.992 | 0.995 | 0.959 | 0.986 | 0.999 |
| $\phi_{m=4}^{\left( J.daily \right)}$ | 2000 | 0.991 | 0.994 | 0.996 | 0.993 | 0.998 | 1 |
| $\phi_{m=4}^{\left( J.daily \right)}$ | 2001 | 0.987 | 0.991 | 0.994 | 0.96 | 0.977 | 0.993 |
| $\phi_{m=4}^{\left( J.daily \right)}$ | 2002 | 0.989 | 0.993 | 0.995 | 0.978 | 0.994 | 0.999 |
| $\phi_{m=4}^{\left( J.daily \right)}$ | 2003 | 0.988 | 0.992 | 0.995 | 0.965 | 0.983 | 0.997 |
| $\phi_{m=4}^{\left( J.daily \right)}$ | 2004 | 0.99 | 0.993 | 0.995 | 0.984 | 0.996 | 0.999 |
| $\phi_{m=4}^{\left( J.daily \right)}$ | 2005 | 0.991 | 0.994 | 0.996 | 0.992 | 0.998 | 1 |
| $\phi_{m=4}^{\left( J.daily \right)}$ | 2006 | 0.989 | 0.992 | 0.995 | 0.97 | 0.99 | 0.999 |
| $\phi_{m=4}^{\left( J.daily \right)}$ | 2007 | 0.99 | 0.993 | 0.995 | 0.988 | 0.997 | 1 |
| $\phi_{m=4}^{\left( J.daily \right)}$ | 2008 | 0.988 | 0.992 | 0.995 | 0.972 | 0.991 | 0.999 |
| $\phi_{m=4}^{\left( J.daily \right)}$ | 2009 | 0.99 | 0.993 | 0.995 | 0.983 | 0.995 | 0.999 |
| $\phi_{m=4}^{\left( J.daily \right)}$ | 2010 | 0.989 | 0.993 | 0.995 | 0.966 | 0.992 | 0.999 |
| $\phi_{m=4}^{\left( J.daily \right)}$ | 2011 | 0.988 | 0.992 | 0.995 | 0.949 | 0.97 | 0.991 |
| $\phi_{m=4}^{\left( J.daily \right)}$ | 2012 | 0.988 | 0.992 | 0.995 | 0.963 | 0.984 | 0.998 |
| $\phi_{m=4}^{\left( J.daily \right)}$ | 2013 | 0.989 | 0.993 | 0.995 | 0.978 | 0.995 | 0.999 |
| $\phi_{m=4}^{\left( J.daily \right)}$ | 2014 | 0.989 | 0.993 | 0.995 | 0.943 | 0.991 | 0.999 |
| $\phi_{m=4}^{\left( J.daily \right)}$ | 2015 | 0.99 | 0.993 | 0.995 | 0.984 | 0.996 | 0.999 |
| $\phi_{m=4}^{\left( J.daily \right)}$ | 2016 | 0.989 | 0.993 | 0.995 | 0.972 | 0.994 | 0.999 |
| $\phi_{m=4}^{\left( J.daily \right)}$ | 2017 | 0.989 | 0.993 | 0.995 | 0.977 | 0.995 | 0.999 |
| $\phi_{m=4}^{\left( J.daily \right)}$ | 2018 | 0.989 | 0.993 | 0.995 | 0.935 | 0.988 | 0.999 |
| $\phi_{m=4}^{\left( J.daily \right)}$ | 2019 | 0.99 | 0.993 | 0.995 | 0.983 | 0.996 | 0.999 |
| $\phi_{m=4}^{\left( J.daily \right)}$ | 2020 | 0.988 | 0.992 | 0.995 | 0.929 | 0.966 | 0.996 |
| $\phi_{m=4}^{\left( J.daily \right)}$ | 2021 | 0.989 | 0.993 | 0.995 | 0.957 | 0.988 | 0.999 |
| $\phi_{m=4}^{\left( J.daily \right)}$ | 2022 | 0.989 | 0.993 | 0.995 | 0.951 | 0.987 | 0.999 |
| ${\phi.\mu}^{(B)}$ | NA | 0.86 | 0.88 | 0.92 | 0.86 | 0.88 | 0.91 |
| $\phi^{(B)}$ | 1998 | 0.81 | 0.85 | 0.89 | 0.83 | 0.86 | 0.89 |
| $\phi^{(B)}$ | 1999 | 0.84 | 0.87 | 0.89 | 0.84 | 0.87 | 0.9 |
| $\phi^{(B)}$ | 2000 | 0.82 | 0.85 | 0.88 | 0.82 | 0.85 | 0.88 |
| $\phi^{(B)}$ | 2001 | 0.85 | 0.87 | 0.9 | 0.85 | 0.88 | 0.9 |
| $\phi^{(B)}$ | 2002 | 0.89 | 0.91 | 0.93 | 0.89 | 0.91 | 0.93 |
| $\phi^{(B)}$ | 2003 | 0.85 | 0.87 | 0.89 | 0.84 | 0.87 | 0.89 |
| $\phi^{(B)}$ | 2004 | 0.86 | 0.88 | 0.9 | 0.85 | 0.88 | 0.9 |
| $\phi^{(B)}$ | 2005 | 0.84 | 0.87 | 0.89 | 0.84 | 0.86 | 0.88 |
| $\phi^{(B)}$ | 2006 | 0.84 | 0.86 | 0.88 | 0.83 | 0.85 | 0.88 |
| $\phi^{(B)}$ | 2007 | 0.81 | 0.84 | 0.87 | 0.8 | 0.83 | 0.86 |
| $\phi^{(B)}$ | 2008 | 0.83 | 0.85 | 0.88 | 0.82 | 0.85 | 0.87 |
| $\phi^{(B)}$ | 2009 | 0.81 | 0.84 | 0.87 | 0.8 | 0.83 | 0.86 |
| $\phi^{(B)}$ | 2010 | 0.82 | 0.85 | 0.87 | 0.81 | 0.84 | 0.87 |
| $\phi^{(B)}$ | 2011 | 0.78 | 0.81 | 0.84 | 0.77 | 0.8 | 0.83 |
| $\phi^{(B)}$ | 2012 | 0.85 | 0.88 | 0.9 | 0.85 | 0.87 | 0.9 |
| $\phi^{(B)}$ | 2013 | 0.85 | 0.87 | 0.9 | 0.84 | 0.86 | 0.89 |
| $\phi^{(B)}$ | 2014 | 0.76 | 0.8 | 0.83 | 0.74 | 0.78 | 0.81 |
| $\phi^{(B)}$ | 2015 | 0.89 | 0.91 | 0.93 | 0.88 | 0.9 | 0.92 |
| $\phi^{(B)}$ | 2016 | 0.86 | 0.88 | 0.91 | 0.84 | 0.87 | 0.9 |
| $\phi^{(B)}$ | 2017 | 0.85 | 0.87 | 0.89 | 0.83 | 0.86 | 0.88 |
| $\phi^{(B)}$ | 2018 | 0.85 | 0.88 | 0.9 | 0.83 | 0.86 | 0.88 |
| $\phi^{(B)}$ | 2019 | 0.87 | 0.9 | 0.92 | 0.86 | 0.89 | 0.91 |
| $\phi^{(B)}$ | 2020 | 0.83 | 0.86 | 0.88 | 0.82 | 0.85 | 0.87 |
| $\phi^{(B)}$ | 2021 | 0.88 | 0.9 | 0.92 | 0.87 | 0.89 | 0.91 |
| $\phi^{(B)}$ | 2022 | 0.87 | 0.89 | 0.91 | 0.86 | 0.88 | 0.9 |
| ${\phi.\mu}_{a=1,s=1}^{\left( NB \right)}$ | NA | 0.92 | 0.93 | 0.95 | 0.92 | 0.93 | 0.94 |
| $\phi_{a=1,s=1}^{\left( NB \right)}$ | 1998 | 0.91 | 0.93 | 0.95 | 0.91 | 0.93 | 0.95 |
| $\phi_{a=1,s=1}^{\left( NB \right)}$ | 1999 | 0.89 | 0.93 | 0.94 | 0.88 | 0.92 | 0.94 |
| $\phi_{a=1,s=1}^{\left( NB \right)}$ | 2000 | 0.9 | 0.93 | 0.95 | 0.9 | 0.92 | 0.94 |
| $\phi_{a=1,s=1}^{\left( NB \right)}$ | 2001 | 0.91 | 0.93 | 0.94 | 0.9 | 0.92 | 0.94 |
| $\phi_{a=1,s=1}^{\left( NB \right)}$ | 2002 | 0.91 | 0.93 | 0.94 | 0.9 | 0.92 | 0.94 |
| $\phi_{a=1,s=1}^{\left( NB \right)}$ | 2003 | 0.9 | 0.93 | 0.94 | 0.9 | 0.92 | 0.94 |
| $\phi_{a=1,s=1}^{\left( NB \right)}$ | 2004 | 0.9 | 0.93 | 0.94 | 0.9 | 0.92 | 0.94 |
| $\phi_{a=1,s=1}^{\left( NB \right)}$ | 2005 | 0.91 | 0.93 | 0.95 | 0.91 | 0.93 | 0.95 |
| $\phi_{a=1,s=1}^{\left( NB \right)}$ | 2006 | 0.91 | 0.93 | 0.95 | 0.91 | 0.93 | 0.95 |
| $\phi_{a=1,s=1}^{\left( NB \right)}$ | 2007 | 0.91 | 0.93 | 0.95 | 0.91 | 0.93 | 0.95 |
| $\phi_{a=1,s=1}^{\left( NB \right)}$ | 2008 | 0.9 | 0.93 | 0.94 | 0.89 | 0.92 | 0.94 |
| $\phi_{a=1,s=1}^{\left( NB \right)}$ | 2009 | 0.9 | 0.93 | 0.94 | 0.89 | 0.92 | 0.94 |
| $\phi_{a=1,s=1}^{\left( NB \right)}$ | 2010 | 0.9 | 0.93 | 0.94 | 0.89 | 0.92 | 0.94 |
| $\phi_{a=1,s=1}^{\left( NB \right)}$ | 2011 | 0.9 | 0.93 | 0.95 | 0.9 | 0.93 | 0.94 |
| $\phi_{a=1,s=1}^{\left( NB \right)}$ | 2012 | 0.91 | 0.93 | 0.95 | 0.91 | 0.93 | 0.95 |
| $\phi_{a=1,s=1}^{\left( NB \right)}$ | 2013 | 0.9 | 0.93 | 0.94 | 0.89 | 0.92 | 0.94 |
| $\phi_{a=1,s=1}^{\left( NB \right)}$ | 2014 | 0.91 | 0.93 | 0.95 | 0.9 | 0.92 | 0.94 |
| $\phi_{a=1,s=1}^{\left( NB \right)}$ | 2015 | 0.91 | 0.93 | 0.95 | 0.9 | 0.93 | 0.94 |
| $\phi_{a=1,s=1}^{\left( NB \right)}$ | 2016 | 0.9 | 0.93 | 0.94 | 0.89 | 0.92 | 0.94 |
| $\phi_{a=1,s=1}^{\left( NB \right)}$ | 2017 | 0.9 | 0.93 | 0.95 | 0.9 | 0.92 | 0.94 |
| $\phi_{a=1,s=1}^{\left( NB \right)}$ | 2018 | 0.9 | 0.93 | 0.94 | 0.9 | 0.92 | 0.94 |
| $\phi_{a=1,s=1}^{\left( NB \right)}$ | 2019 | 0.9 | 0.93 | 0.94 | 0.89 | 0.92 | 0.94 |
| $\phi_{a=1,s=1}^{\left( NB \right)}$ | 2020 | 0.91 | 0.93 | 0.95 | 0.9 | 0.92 | 0.94 |
| $\phi_{a=1,s=1}^{\left( NB \right)}$ | 2021 | 0.91 | 0.93 | 0.95 | 0.9 | 0.92 | 0.94 |
| $\phi_{a=1,s=1}^{\left( NB \right)}$ | 2022 | 0.91 | 0.93 | 0.95 | 0.9 | 0.92 | 0.95 |
| ${\phi.\mu}_{a=1,s=2}^{\left( NB \right)}$ | NA | 0.91 | 0.93 | 0.95 | 0.9 | 0.92 | 0.94 |
| $\phi_{a=1,s=2}^{\left( NB \right)}$ | 1998 | 0.85 | 0.91 | 0.95 | 0.85 | 0.91 | 0.95 |
| $\phi_{a=1,s=2}^{\left( NB \right)}$ | 1999 | 0.71 | 0.8 | 0.87 | 0.73 | 0.81 | 0.88 |
| $\phi_{a=1,s=2}^{\left( NB \right)}$ | 2000 | 0.88 | 0.93 | 0.97 | 0.87 | 0.93 | 0.96 |
| $\phi_{a=1,s=2}^{\left( NB \right)}$ | 2001 | 0.83 | 0.9 | 0.95 | 0.82 | 0.89 | 0.94 |
| $\phi_{a=1,s=2}^{\left( NB \right)}$ | 2002 | 0.88 | 0.92 | 0.95 | 0.88 | 0.92 | 0.95 |
| $\phi_{a=1,s=2}^{\left( NB \right)}$ | 2003 | 0.87 | 0.91 | 0.95 | 0.86 | 0.91 | 0.94 |
| $\phi_{a=1,s=2}^{\left( NB \right)}$ | 2004 | 0.87 | 0.92 | 0.95 | 0.85 | 0.9 | 0.94 |
| $\phi_{a=1,s=2}^{\left( NB \right)}$ | 2005 | 0.83 | 0.89 | 0.93 | 0.83 | 0.88 | 0.92 |
| $\phi_{a=1,s=2}^{\left( NB \right)}$ | 2006 | 0.88 | 0.92 | 0.95 | 0.87 | 0.91 | 0.94 |
| $\phi_{a=1,s=2}^{\left( NB \right)}$ | 2007 | 0.91 | 0.95 | 0.98 | 0.9 | 0.94 | 0.97 |
| $\phi_{a=1,s=2}^{\left( NB \right)}$ | 2008 | 0.9 | 0.94 | 0.97 | 0.89 | 0.93 | 0.96 |
| $\phi_{a=1,s=2}^{\left( NB \right)}$ | 2009 | 0.88 | 0.93 | 0.96 | 0.88 | 0.92 | 0.96 |
| $\phi_{a=1,s=2}^{\left( NB \right)}$ | 2010 | 0.87 | 0.91 | 0.94 | 0.86 | 0.91 | 0.94 |
| $\phi_{a=1,s=2}^{\left( NB \right)}$ | 2011 | 0.91 | 0.95 | 0.97 | 0.9 | 0.94 | 0.97 |
| $\phi_{a=1,s=2}^{\left( NB \right)}$ | 2012 | 0.92 | 0.95 | 0.98 | 0.92 | 0.95 | 0.97 |
| $\phi_{a=1,s=2}^{\left( NB \right)}$ | 2013 | 0.87 | 0.92 | 0.96 | 0.85 | 0.91 | 0.95 |
| $\phi_{a=1,s=2}^{\left( NB \right)}$ | 2014 | 0.91 | 0.95 | 0.97 | 0.9 | 0.94 | 0.97 |
| $\phi_{a=1,s=2}^{\left( NB \right)}$ | 2015 | 0.91 | 0.95 | 0.97 | 0.9 | 0.93 | 0.96 |
| $\phi_{a=1,s=2}^{\left( NB \right)}$ | 2016 | 0.91 | 0.95 | 0.97 | 0.89 | 0.93 | 0.96 |
| $\phi_{a=1,s=2}^{\left( NB \right)}$ | 2017 | 0.9 | 0.95 | 0.97 | 0.88 | 0.93 | 0.96 |
| $\phi_{a=1,s=2}^{\left( NB \right)}$ | 2018 | 0.93 | 0.96 | 0.98 | 0.91 | 0.94 | 0.97 |
| $\phi_{a=1,s=2}^{\left( NB \right)}$ | 2019 | 0.9 | 0.94 | 0.97 | 0.89 | 0.93 | 0.96 |
| $\phi_{a=1,s=2}^{\left( NB \right)}$ | 2020 | 0.89 | 0.93 | 0.96 | 0.86 | 0.92 | 0.95 |
| $\phi_{a=1,s=2}^{\left( NB \right)}$ | 2021 | 0.82 | 0.88 | 0.92 | 0.8 | 0.86 | 0.91 |
| $\phi_{a=1,s=2}^{\left( NB \right)}$ | 2022 | 0.84 | 0.9 | 0.95 | 0.84 | 0.9 | 0.94 |
| ${\phi.\mu}_{a=2,s=1}^{\left( NB \right)}$ | NA | 0.91 | 0.92 | 0.94 | 0.9 | 0.91 | 0.93 |
| $\phi_{a=2,s=1}^{\left( NB \right)}$ | 1998 | 0.9 | 0.92 | 0.95 | 0.89 | 0.92 | 0.94 |
| $\phi_{a=2,s=1}^{\left( NB \right)}$ | 1999 | 0.84 | 0.9 | 0.92 | 0.81 | 0.88 | 0.91 |
| $\phi_{a=2,s=1}^{\left( NB \right)}$ | 2000 | 0.9 | 0.92 | 0.95 | 0.89 | 0.92 | 0.94 |
| $\phi_{a=2,s=1}^{\left( NB \right)}$ | 2001 | 0.9 | 0.92 | 0.95 | 0.9 | 0.92 | 0.95 |
| $\phi_{a=2,s=1}^{\left( NB \right)}$ | 2002 | 0.88 | 0.91 | 0.94 | 0.87 | 0.9 | 0.93 |
| $\phi_{a=2,s=1}^{\left( NB \right)}$ | 2003 | 0.9 | 0.92 | 0.95 | 0.9 | 0.92 | 0.95 |
| $\phi_{a=2,s=1}^{\left( NB \right)}$ | 2004 | 0.87 | 0.91 | 0.93 | 0.86 | 0.9 | 0.93 |
| $\phi_{a=2,s=1}^{\left( NB \right)}$ | 2005 | 0.87 | 0.91 | 0.93 | 0.86 | 0.9 | 0.92 |
| $\phi_{a=2,s=1}^{\left( NB \right)}$ | 2006 | 0.87 | 0.91 | 0.93 | 0.85 | 0.89 | 0.92 |
| $\phi_{a=2,s=1}^{\left( NB \right)}$ | 2007 | 0.89 | 0.92 | 0.94 | 0.87 | 0.91 | 0.93 |
| $\phi_{a=2,s=1}^{\left( NB \right)}$ | 2008 | 0.86 | 0.9 | 0.92 | 0.83 | 0.88 | 0.91 |
| $\phi_{a=2,s=1}^{\left( NB \right)}$ | 2009 | 0.89 | 0.91 | 0.94 | 0.87 | 0.9 | 0.93 |
| $\phi_{a=2,s=1}^{\left( NB \right)}$ | 2010 | 0.89 | 0.92 | 0.94 | 0.87 | 0.91 | 0.94 |
| $\phi_{a=2,s=1}^{\left( NB \right)}$ | 2011 | 0.87 | 0.91 | 0.93 | 0.86 | 0.9 | 0.93 |
| $\phi_{a=2,s=1}^{\left( NB \right)}$ | 2012 | 0.9 | 0.92 | 0.95 | 0.89 | 0.92 | 0.95 |
| $\phi_{a=2,s=1}^{\left( NB \right)}$ | 2013 | 0.89 | 0.92 | 0.94 | 0.87 | 0.91 | 0.93 |
| $\phi_{a=2,s=1}^{\left( NB \right)}$ | 2014 | 0.88 | 0.92 | 0.94 | 0.86 | 0.9 | 0.93 |
| $\phi_{a=2,s=1}^{\left( NB \right)}$ | 2015 | 0.88 | 0.91 | 0.93 | 0.86 | 0.9 | 0.93 |
| $\phi_{a=2,s=1}^{\left( NB \right)}$ | 2016 | 0.9 | 0.92 | 0.95 | 0.89 | 0.92 | 0.95 |
| $\phi_{a=2,s=1}^{\left( NB \right)}$ | 2017 | 0.89 | 0.92 | 0.94 | 0.89 | 0.91 | 0.94 |
| $\phi_{a=2,s=1}^{\left( NB \right)}$ | 2018 | 0.9 | 0.92 | 0.95 | 0.88 | 0.92 | 0.95 |
| $\phi_{a=2,s=1}^{\left( NB \right)}$ | 2019 | 0.9 | 0.92 | 0.95 | 0.89 | 0.92 | 0.95 |
| $\phi_{a=2,s=1}^{\left( NB \right)}$ | 2020 | 0.89 | 0.92 | 0.94 | 0.88 | 0.91 | 0.94 |
| $\phi_{a=2,s=1}^{\left( NB \right)}$ | 2021 | 0.89 | 0.92 | 0.94 | 0.88 | 0.91 | 0.94 |
| $\phi_{a=2,s=1}^{\left( NB \right)}$ | 2022 | 0.9 | 0.93 | 0.96 | 0.9 | 0.93 | 0.96 |
| ${\phi.\mu}_{a=2,s=2}^{\left( NB \right)}$ | NA | 0.92 | 0.94 | 0.95 | 0.9 | 0.92 | 0.94 |
| $\phi_{a=2,s=2}^{\left( NB \right)}$ | 1998 | 0.89 | 0.93 | 0.95 | 0.88 | 0.91 | 0.94 |
| $\phi_{a=2,s=2}^{\left( NB \right)}$ | 1999 | 0.79 | 0.85 | 0.91 | 0.74 | 0.82 | 0.88 |
| $\phi_{a=2,s=2}^{\left( NB \right)}$ | 2000 | 0.86 | 0.9 | 0.93 | 0.83 | 0.87 | 0.91 |
| $\phi_{a=2,s=2}^{\left( NB \right)}$ | 2001 | 0.89 | 0.92 | 0.94 | 0.87 | 0.9 | 0.93 |
| $\phi_{a=2,s=2}^{\left( NB \right)}$ | 2002 | 0.89 | 0.92 | 0.95 | 0.87 | 0.91 | 0.94 |
| $\phi_{a=2,s=2}^{\left( NB \right)}$ | 2003 | 0.9 | 0.93 | 0.95 | 0.88 | 0.91 | 0.94 |
| $\phi_{a=2,s=2}^{\left( NB \right)}$ | 2004 | 0.87 | 0.91 | 0.93 | 0.84 | 0.88 | 0.91 |
| $\phi_{a=2,s=2}^{\left( NB \right)}$ | 2005 | 0.9 | 0.93 | 0.95 | 0.87 | 0.9 | 0.93 |
| $\phi_{a=2,s=2}^{\left( NB \right)}$ | 2006 | 0.87 | 0.91 | 0.94 | 0.83 | 0.88 | 0.92 |
| $\phi_{a=2,s=2}^{\left( NB \right)}$ | 2007 | 0.9 | 0.93 | 0.95 | 0.88 | 0.92 | 0.94 |
| $\phi_{a=2,s=2}^{\left( NB \right)}$ | 2008 | 0.9 | 0.93 | 0.95 | 0.88 | 0.91 | 0.94 |
| $\phi_{a=2,s=2}^{\left( NB \right)}$ | 2009 | 0.88 | 0.92 | 0.95 | 0.86 | 0.9 | 0.94 |
| $\phi_{a=2,s=2}^{\left( NB \right)}$ | 2010 | 0.9 | 0.93 | 0.96 | 0.88 | 0.92 | 0.95 |
| $\phi_{a=2,s=2}^{\left( NB \right)}$ | 2011 | 0.9 | 0.93 | 0.95 | 0.87 | 0.9 | 0.93 |
| $\phi_{a=2,s=2}^{\left( NB \right)}$ | 2012 | 0.94 | 0.96 | 0.98 | 0.91 | 0.94 | 0.97 |
| $\phi_{a=2,s=2}^{\left( NB \right)}$ | 2013 | 0.9 | 0.94 | 0.96 | 0.88 | 0.92 | 0.95 |
| $\phi_{a=2,s=2}^{\left( NB \right)}$ | 2014 | 0.89 | 0.92 | 0.95 | 0.85 | 0.89 | 0.93 |
| $\phi_{a=2,s=2}^{\left( NB \right)}$ | 2015 | 0.94 | 0.97 | 0.98 | 0.92 | 0.95 | 0.97 |
| $\phi_{a=2,s=2}^{\left( NB \right)}$ | 2016 | 0.93 | 0.95 | 0.97 | 0.9 | 0.93 | 0.96 |
| $\phi_{a=2,s=2}^{\left( NB \right)}$ | 2017 | 0.93 | 0.95 | 0.97 | 0.9 | 0.93 | 0.96 |
| $\phi_{a=2,s=2}^{\left( NB \right)}$ | 2018 | 0.87 | 0.91 | 0.94 | 0.83 | 0.88 | 0.92 |
| $\phi_{a=2,s=2}^{\left( NB \right)}$ | 2019 | 0.93 | 0.95 | 0.97 | 0.91 | 0.93 | 0.96 |
| $\phi_{a=2,s=2}^{\left( NB \right)}$ | 2020 | 0.95 | 0.97 | 0.98 | 0.93 | 0.95 | 0.97 |
| $\phi_{a=2,s=2}^{\left( NB \right)}$ | 2021 | 0.89 | 0.93 | 0.95 | 0.85 | 0.89 | 0.93 |
| $\phi_{a=2,s=2}^{\left( NB \right)}$ | 2022 | 0.91 | 0.94 | 0.97 | 0.89 | 0.93 | 0.96 |
| ${P.\mu}_{m=1,s=1}$ | NA | 0.24 | 0.64 | 1.34 | 0.11 | 0.39 | 0.94 |
| $P_{m=1,s=1}$ | 1998 | 1.316 | 1.82 | 2.43 | 1.243 | 1.74 | 2.35 |
| $P_{m=1,s=1}$ | 1999 | 0.743 | 1.13 | 1.6 | 0.616 | 0.98 | 1.45 |
| $P_{m=1,s=1}$ | 2000 | 1.297 | 1.88 | 2.33 | 0.706 | 1.24 | 1.91 |
| $P_{m=1,s=1}$ | 2001 | 0.661 | 0.95 | 1.32 | 0.595 | 0.87 | 1.22 |
| $P_{m=1,s=1}$ | 2002 | 0.001 | 0.02 | 0.09 | 0 | 0.01 | 0.07 |
| $P_{m=1,s=1}$ | 2003 | 0.641 | 1.02 | 1.36 | 0.428 | 0.78 | 1.19 |
| $P_{m=1,s=1}$ | 2004 | 1.052 | 1.54 | 2.01 | 0.964 | 1.45 | 1.96 |
| $P_{m=1,s=1}$ | 2005 | 0.002 | 0.05 | 0.25 | 0 | 0.02 | 0.14 |
| $P_{m=1,s=1}$ | 2006 | 0.002 | 0.04 | 0.2 | 0 | 0.02 | 0.14 |
| $P_{m=1,s=1}$ | 2007 | 0.53 | 0.95 | 1.51 | 0.404 | 0.76 | 1.25 |
| $P_{m=1,s=1}$ | 2008 | 0.349 | 0.84 | 1.61 | 0.223 | 0.59 | 1.23 |
| $P_{m=1,s=1}$ | 2009 | 1.056 | 1.64 | 2.1 | 0.734 | 1.32 | 1.86 |
| $P_{m=1,s=1}$ | 2010 | 1.108 | 1.65 | 2.12 | 0.751 | 1.27 | 1.84 |
| $P_{m=1,s=1}$ | 2011 | 0.005 | 0.18 | 1.1 | 0.001 | 0.04 | 0.37 |
| $P_{m=1,s=1}$ | 2012 | 0.005 | 0.26 | 1.78 | 0.001 | 0.06 | 0.56 |
| $P_{m=1,s=1}$ | 2013 | 0.001 | 0.04 | 0.2 | 0 | 0.02 | 0.15 |
| $P_{m=1,s=1}$ | 2014 | 0.474 | 1.15 | 1.55 | 0.001 | 0.21 | 1.06 |
| $P_{m=1,s=1}$ | 2015 | 0.632 | 1.2 | 1.75 | 0.383 | 0.81 | 1.37 |
| $P_{m=1,s=1}$ | 2016 | 0.42 | 1.03 | 1.54 | 0.125 | 0.62 | 1.18 |
| $P_{m=1,s=1}$ | 2017 | 0.002 | 0.05 | 0.31 | 0 | 0.03 | 0.23 |
| $P_{m=1,s=1}$ | 2018 | 0.002 | 0.08 | 0.44 | 0 | 0.03 | 0.21 |
| $P_{m=1,s=1}$ | 2019 | 0.002 | 0.07 | 0.43 | 0 | 0.03 | 0.21 |
| $P_{m=1,s=1}$ | 2020 | 0.003 | 0.1 | 0.59 | 0 | 0.02 | 0.18 |
| $P_{m=1,s=1}$ | 2021 | 0.383 | 0.74 | 1.2 | 0.267 | 0.52 | 0.89 |
| $P_{m=1,s=1}$ | 2022 | 0.168 | 0.4 | 0.7 | 0.083 | 0.27 | 0.52 |
| ${P.\mu}_{m=2,s=1}$ | NA | 0.03 | 0.15 | 0.47 | 0.01 | 0.09 | 0.33 |
| $P_{m=2,s=1}$ | 1998 | 0.26 | 0.55 | 0.98 | 0.22 | 0.48 | 0.86 |
| $P_{m=2,s=1}$ | 1999 | 0.7 | 1.17 | 1.74 | 0.58 | 1.01 | 1.61 |
| $P_{m=2,s=1}$ | 2000 | 0.61 | 1.17 | 2.04 | 0.64 | 1.36 | 2.36 |
| $P_{m=2,s=1}$ | 2001 | 0.0001 | 0.01 | 0.14 | <0.0001 | 0.01 | 0.08 |
| $P_{m=2,s=1}$ | 2002 | 0.0001 | 0.01 | 0.11 | <0.0001 | 0.004 | 0.06 |
| $P_{m=2,s=1}$ | 2003 | 1.21 | 1.69 | 2.07 | 1.09 | 1.59 | 2.01 |
| $P_{m=2,s=1}$ | 2004 | 0.0001 | 0.04 | 0.5 | <0.0001 | 0.01 | 0.18 |
| $P_{m=2,s=1}$ | 2005 | 0.0001 | 0.02 | 0.19 | <0.0001 | 0.01 | 0.12 |
| $P_{m=2,s=1}$ | 2006 | 0.0001 | 0.04 | 0.4 | <0.0001 | 0.02 | 0.25 |
| $P_{m=2,s=1}$ | 2007 | 0.0001 | 0.02 | 0.21 | <0.0001 | 0.01 | 0.15 |
| $P_{m=2,s=1}$ | 2008 | 1.14 | 1.59 | 1.97 | 0.87 | 1.37 | 1.76 |
| $P_{m=2,s=1}$ | 2009 | 0.0001 | 0.02 | 0.28 | <0.0001 | 0.01 | 0.26 |
| $P_{m=2,s=1}$ | 2010 | 0.0001 | 0.02 | 0.2 | <0.0001 | 0.01 | 0.16 |
| $P_{m=2,s=1}$ | 2011 | 0.0001 | 0.03 | 0.47 | <0.0001 | 0.01 | 0.3 |
| $P_{m=2,s=1}$ | 2012 | 0.0003 | 0.14 | 0.97 | <0.0001 | 0.02 | 0.52 |
| $P_{m=2,s=1}$ | 2013 | 0.0001 | 0.01 | 0.17 | <0.0001 | 0.01 | 0.12 |
| $P_{m=2,s=1}$ | 2014 | 0.0002 | 0.05 | 0.81 | <0.0001 | 0.02 | 0.39 |
| $P_{m=2,s=1}$ | 2015 | 0.0001 | 0.01 | 0.15 | <0.0001 | 0.01 | 0.1 |
| $P_{m=2,s=1}$ | 2016 | 0.0001 | 0.03 | 0.38 | <0.0001 | 0.01 | 0.24 |
| $P_{m=2,s=1}$ | 2017 | 0.06 | 0.35 | 1.07 | 0.03 | 0.22 | 0.71 |
| $P_{m=2,s=1}$ | 2018 | 0.38 | 0.83 | 1.52 | 0.32 | 0.74 | 1.41 |
| $P_{m=2,s=1}$ | 2019 | 0.78 | 1.08 | 1.35 | 0.63 | 0.92 | 1.21 |
| $P_{m=2,s=1}$ | 2020 | 0.0001 | 0.04 | 0.55 | <0.0001 | 0.01 | 0.21 |
| $P_{m=2,s=1}$ | 2021 | 0.07 | 0.23 | 0.52 | 0.06 | 0.18 | 0.43 |
| $P_{m=2,s=1}$ | 2022 | <0.0001 | 0.01 | 0.06 | <0.0001 | 0.003 | 0.05 |
| ${P.\mu}_{m=3,s=1}$ | NA | 0.01 | 0.07 | 0.26 | 0.004 | 0.05 | 0.22 |
| $P_{m=3,s=1}$ | 1998 | <0.0001 | 0.01 | 0.12 | <0.0001 | 0.01 | 0.13 |
| $P_{m=3,s=1}$ | 1999 | <0.0001 | 0.03 | 0.37 | <0.0001 | 0.01 | 0.24 |
| $P_{m=3,s=1}$ | 2000 | 0.25 | 0.72 | 1.45 | 0.21 | 0.63 | 1.56 |
| $P_{m=3,s=1}$ | 2001 | 0.41 | 0.69 | 1.07 | 0.36 | 0.63 | 1.02 |
| $P_{m=3,s=1}$ | 2002 | 0.0001 | 0.11 | 0.52 | <0.0001 | 0.08 | 0.55 |
| $P_{m=3,s=1}$ | 2003 | <0.0001 | 0.01 | 0.22 | <0.0001 | 0.01 | 0.14 |
| $P_{m=3,s=1}$ | 2004 | <0.0001 | 0.01 | 0.11 | <0.0001 | 0.01 | 0.12 |
| $P_{m=3,s=1}$ | 2005 | <0.0001 | 0.02 | 0.34 | <0.0001 | 0.01 | 0.22 |
| $P_{m=3,s=1}$ | 2006 | <0.0001 | 0.02 | 0.27 | <0.0001 | 0.01 | 0.17 |
| $P_{m=3,s=1}$ | 2007 | 0.76 | 1.09 | 1.45 | 0.71 | 1.04 | 1.45 |
| $P_{m=3,s=1}$ | 2008 | 0.0001 | 0.11 | 0.89 | 0.0001 | 0.07 | 0.8 |
| $P_{m=3,s=1}$ | 2009 | 0.0001 | 0.24 | 1.19 | <0.0001 | 0.03 | 0.67 |
| $P_{m=3,s=1}$ | 2010 | <0.0001 | 0.01 | 0.21 | <0.0001 | 0.02 | 0.43 |
| $P_{m=3,s=1}$ | 2011 | 0.03 | 1.11 | 1.7 | 0.0001 | 0.16 | 1.27 |
| $P_{m=3,s=1}$ | 2012 | <0.0001 | 0.02 | 0.27 | <0.0001 | 0.01 | 0.29 |
| $P_{m=3,s=1}$ | 2013 | <0.0001 | 0.03 | 0.49 | <0.0001 | 0.02 | 0.31 |
| $P_{m=3,s=1}$ | 2014 | <0.0001 | 0.02 | 0.33 | <0.0001 | 0.02 | 0.41 |
| $P_{m=3,s=1}$ | 2015 | 0.33 | 0.62 | 0.9 | 0.3 | 0.56 | 0.84 |
| $P_{m=3,s=1}$ | 2016 | <0.0001 | 0.02 | 0.28 | <0.0001 | 0.01 | 0.22 |
| $P_{m=3,s=1}$ | 2017 | 0.0003 | 0.29 | 0.65 | 0.0001 | 0.17 | 0.56 |
| $P_{m=3,s=1}$ | 2018 | <0.0001 | 0.02 | 0.27 | <0.0001 | 0.01 | 0.19 |
| $P_{m=3,s=1}$ | 2019 | <0.0001 | 0.01 | 0.18 | <0.0001 | 0.01 | 0.18 |
| $P_{m=3,s=1}$ | 2020 | <0.0001 | 0.01 | 0.12 | <0.0001 | 0.01 | 0.15 |
| $P_{m=3,s=1}$ | 2021 | <0.0001 | 0.01 | 0.09 | <0.0001 | 0.01 | 0.08 |
| $P_{m=3,s=1}$ | 2022 | <0.0001 | 0.01 | 0.07 | <0.0001 | 0.01 | 0.06 |
| ${P.\mu}_{m=4,s=1}$ | NA | 0.0003 | 0.01 | 0.04 | 0.002 | 0.02 | 0.1 |
| $P_{m=4,s=1}$ | 1998 | 0.002 | 0.03 | 0.19 | 0.0034 | 0.05 | 0.23 |
| $P_{m=4,s=1}$ | 1999 | <0.0001 | 0.002 | 0.05 | <0.0001 | 0.009 | 0.01 |
| $P_{m=4,s=1}$ | 2000 | 0.36 | 0.79 | 1.05 | 0.49 | 0.837 | 0.84 |
| $P_{m=4,s=1}$ | 2001 | <0.0001 | 0.002 | 0.04 | <0.0001 | 0.007 | 0.01 |
| $P_{m=4,s=1}$ | 2002 | <0.0001 | 0.002 | 0.05 | <0.0001 | 0.006 | 0.01 |
| $P_{m=4,s=1}$ | 2003 | <0.0001 | 0.003 | 0.06 | <0.0001 | 0.009 | 0.01 |
| $P_{m=4,s=1}$ | 2004 | <0.0001 | 0.01 | 0.11 | 0.0001 | 0.036 | 0.04 |
| $P_{m=4,s=1}$ | 2005 | <0.0001 | 0.01 | 0.56 | 0.0003 | 0.29 | 0.29 |
| $P_{m=4,s=1}$ | 2006 | <0.0001 | 0.003 | 0.06 | <0.0001 | 0.009 | 0.01 |
| $P_{m=4,s=1}$ | 2007 | <0.0001 | 0.01 | 0.92 | 0.0002 | 0.163 | 0.16 |
| $P_{m=4,s=1}$ | 2008 | <0.0001 | 0.003 | 0.09 | <0.0001 | 0.013 | 0.01 |
| $P_{m=4,s=1}$ | 2009 | <0.0001 | 0.01 | 0.2 | <0.0001 | 0.013 | 0.01 |
| $P_{m=4,s=1}$ | 2010 | <0.0001 | 0.004 | 0.08 | <0.0001 | 0.01 | 0.01 |
| $P_{m=4,s=1}$ | 2011 | <0.0001 | 0.004 | 0.13 | <0.0001 | 0.015 | 0.02 |
| $P_{m=4,s=1}$ | 2012 | <0.0001 | 0.003 | 0.09 | <0.0001 | 0.011 | 0.01 |
| $P_{m=4,s=1}$ | 2013 | <0.0001 | 0.004 | 0.13 | <0.0001 | 0.014 | 0.01 |
| $P_{m=4,s=1}$ | 2014 | <0.0001 | 0.003 | 0.05 | <0.0001 | 0.009 | 0.01 |
| $P_{m=4,s=1}$ | 2015 | <0.0001 | 0.003 | 0.1 | <0.0001 | 0.01 | 0.01 |
| $P_{m=4,s=1}$ | 2016 | <0.0001 | 0.004 | 0.2 | <0.0001 | 0.012 | 0.01 |
| $P_{m=4,s=1}$ | 2017 | <0.0001 | 0.01 | 0.12 | <0.0001 | 0.013 | 0.01 |
| $P_{m=4,s=1}$ | 2018 | <0.0001 | 0.003 | 0.07 | <0.0001 | 0.007 | 0.01 |
| $P_{m=4,s=1}$ | 2019 | <0.0001 | 0.01 | 0.12 | 0.0001 | 0.04 | 0.04 |
| $P_{m=4,s=1}$ | 2020 | <0.0001 | 0.002 | 0.04 | <0.0001 | 0.007 | 0.01 |
| $P_{m=4,s=1}$ | 2021 | <0.0001 | 0.002 | 0.05 | <0.0001 | 0.006 | 0.01 |
| $P_{m=4,s=1}$ | 2022 | <0.0001 | 0.002 | 0.03 | <0.0001 | 0.005 | 0.01 |
| ${P.\mu}_{m=1,s=2}$ | NA | 2.87 | 3.88 | 5.59 | 2.64 | 3.84 | 5.15 |
| $P_{m=1,s=2}$ | 1998 | 3.09 | 3.65 | 4.3 | 3.02 | 3.6 | 4.23 |
| $P_{m=1,s=2}$ | 1999 | 1.72 | 2 | 2.31 | 1.65 | 1.93 | 2.24 |
| $P_{m=1,s=2}$ | 2000 | 1.9 | 2.22 | 2.58 | 1.74 | 2.06 | 2.44 |
| $P_{m=1,s=2}$ | 2001 | 2.89 | 3.33 | 3.82 | 2.76 | 3.2 | 3.67 |
| $P_{m=1,s=2}$ | 2002 | 2.48 | 2.84 | 3.25 | 2.35 | 2.74 | 3.16 |
| $P_{m=1,s=2}$ | 2003 | 1.16 | 1.37 | 1.6 | 1.09 | 1.29 | 1.54 |
| $P_{m=1,s=2}$ | 2004 | 1.82 | 2.08 | 2.37 | 1.78 | 2.06 | 2.34 |
| $P_{m=1,s=2}$ | 2005 | 1.31 | 1.59 | 1.91 | 1.12 | 1.39 | 1.7 |
| $P_{m=1,s=2}$ | 2006 | 2.03 | 2.36 | 2.72 | 1.86 | 2.19 | 2.56 |
| $P_{m=1,s=2}$ | 2007 | 2.76 | 3.17 | 3.63 | 2.61 | 3.01 | 3.46 |
| $P_{m=1,s=2}$ | 2008 | 2.91 | 3.34 | 3.8 | 2.76 | 3.18 | 3.65 |
| $P_{m=1,s=2}$ | 2009 | 1.66 | 1.98 | 2.34 | 1.49 | 1.81 | 2.18 |
| $P_{m=1,s=2}$ | 2010 | 1.74 | 2.06 | 2.41 | 1.58 | 1.9 | 2.25 |
| $P_{m=1,s=2}$ | 2011 | 1.67 | 2.02 | 2.42 | 1.51 | 1.84 | 2.23 |
| $P_{m=1,s=2}$ | 2012 | 3.51 | 3.95 | 4.42 | 3.32 | 3.74 | 4.2 |
| $P_{m=1,s=2}$ | 2013 | 3.39 | 3.81 | 4.26 | 3.23 | 3.65 | 4.11 |
| $P_{m=1,s=2}$ | 2014 | 1.18 | 1.44 | 1.73 | 1.06 | 1.31 | 1.59 |
| $P_{m=1,s=2}$ | 2015 | 1.48 | 1.88 | 2.34 | 1.21 | 1.6 | 2.05 |
| $P_{m=1,s=2}$ | 2016 | 1.3 | 1.6 | 1.94 | 1.12 | 1.42 | 1.74 |
| $P_{m=1,s=2}$ | 2017 | 2.24 | 2.59 | 2.98 | 2.1 | 2.45 | 2.83 |
| $P_{m=1,s=2}$ | 2018 | 0.93 | 1.15 | 1.38 | 0.8 | 0.99 | 1.22 |
| $P_{m=1,s=2}$ | 2019 | 2.14 | 2.53 | 2.94 | 1.9 | 2.27 | 2.68 |
| $P_{m=1,s=2}$ | 2020 | 1.85 | 2.13 | 2.44 | 1.6 | 1.86 | 2.14 |
| $P_{m=1,s=2}$ | 2021 | 1.28 | 1.54 | 1.82 | 1.12 | 1.36 | 1.61 |
| $P_{m=1,s=2}$ | 2022 | 1.63 | 1.9 | 2.2 | 1.44 | 1.69 | 1.98 |
| ${P.\mu}_{m=2,s=2}$ | NA | 2.02 | 2.66 | 3.7 | 1.86 | 2.59 | 3.37 |
| $P_{m=2,s=2}$ | 1998 | 1.38 | 1.77 | 2.24 | 1.29 | 1.67 | 2.14 |
| $P_{m=2,s=2}$ | 1999 | 1.73 | 2.05 | 2.41 | 1.66 | 1.99 | 2.37 |
| $P_{m=2,s=2}$ | 2000 | 2.3 | 2.71 | 3.18 | 2.32 | 2.76 | 3.25 |
| $P_{m=2,s=2}$ | 2001 | 2.21 | 2.56 | 2.96 | 1.99 | 2.34 | 2.73 |
| $P_{m=2,s=2}$ | 2002 | 1.11 | 1.34 | 1.61 | 0.93 | 1.14 | 1.39 |
| $P_{m=2,s=2}$ | 2003 | 1.7 | 1.98 | 2.28 | 1.64 | 1.92 | 2.24 |
| $P_{m=2,s=2}$ | 2004 | 1.3 | 1.57 | 1.85 | 1.16 | 1.39 | 1.64 |
| $P_{m=2,s=2}$ | 2005 | 1.07 | 1.33 | 1.64 | 0.92 | 1.18 | 1.49 |
| $P_{m=2,s=2}$ | 2006 | 0.61 | 0.87 | 1.15 | 0.47 | 0.69 | 0.95 |
| $P_{m=2,s=2}$ | 2007 | 1.23 | 1.53 | 1.89 | 1.13 | 1.42 | 1.76 |
| $P_{m=2,s=2}$ | 2008 | 1.48 | 1.79 | 2.14 | 1.32 | 1.62 | 1.96 |
| $P_{m=2,s=2}$ | 2009 | 2.1 | 2.53 | 3.03 | 2.14 | 2.59 | 3.11 |
| $P_{m=2,s=2}$ | 2010 | 1.81 | 2.21 | 2.67 | 1.77 | 2.21 | 2.72 |
| $P_{m=2,s=2}$ | 2011 | 1.83 | 2.21 | 2.64 | 1.71 | 2.1 | 2.54 |
| $P_{m=2,s=2}$ | 2012 | 1.07 | 1.36 | 1.68 | 0.98 | 1.24 | 1.54 |
| $P_{m=2,s=2}$ | 2013 | 1.39 | 1.67 | 2 | 1.32 | 1.6 | 1.94 |
| $P_{m=2,s=2}$ | 2014 | 1.47 | 1.86 | 2.32 | 1.31 | 1.68 | 2.13 |
| $P_{m=2,s=2}$ | 2015 | 0.84 | 1.07 | 1.34 | 0.75 | 0.98 | 1.25 |
| $P_{m=2,s=2}$ | 2016 | 1 | 1.31 | 1.68 | 0.84 | 1.13 | 1.48 |
| $P_{m=2,s=2}$ | 2017 | 1.51 | 1.83 | 2.18 | 1.39 | 1.7 | 2.06 |
| $P_{m=2,s=2}$ | 2018 | 1.68 | 2 | 2.35 | 1.63 | 1.95 | 2.32 |
| $P_{m=2,s=2}$ | 2019 | 0.99 | 1.21 | 1.47 | 0.85 | 1.08 | 1.35 |
| $P_{m=2,s=2}$ | 2020 | 0.97 | 1.23 | 1.5 | 0.83 | 1.05 | 1.31 |
| $P_{m=2,s=2}$ | 2021 | 1.13 | 1.36 | 1.63 | 1.03 | 1.25 | 1.51 |
| $P_{m=2,s=2}$ | 2022 | 0.84 | 1.05 | 1.29 | 0.75 | 0.96 | 1.22 |
| ${P.\mu}_{m=3,s=2}$ | NA | 1.67 | 2.17 | 2.91 | 1.64 | 2.21 | 2.85 |
| $P_{m=3,s=2}$ | 1998 | 2.45 | 3.07 | 3.78 | 2.49 | 3.14 | 3.94 |
| $P_{m=3,s=2}$ | 1999 | 0.98 | 1.22 | 1.5 | 0.9 | 1.14 | 1.41 |
| $P_{m=3,s=2}$ | 2000 | 1.81 | 2.23 | 2.71 | 1.8 | 2.23 | 2.71 |
| $P_{m=3,s=2}$ | 2001 | 2.03 | 2.42 | 2.86 | 1.95 | 2.36 | 2.82 |
| $P_{m=3,s=2}$ | 2002 | 1.36 | 1.66 | 1.99 | 1.33 | 1.65 | 2.03 |
| $P_{m=3,s=2}$ | 2003 | 1.44 | 1.71 | 2.01 | 1.35 | 1.63 | 1.95 |
| $P_{m=3,s=2}$ | 2004 | 0.8 | 1.03 | 1.31 | 0.76 | 1.05 | 1.41 |
| $P_{m=3,s=2}$ | 2005 | 1.65 | 1.97 | 2.35 | 1.56 | 1.89 | 2.27 |
| $P_{m=3,s=2}$ | 2006 | 1.07 | 1.34 | 1.66 | 0.98 | 1.23 | 1.57 |
| $P_{m=3,s=2}$ | 2007 | 0.94 | 1.23 | 1.59 | 0.88 | 1.18 | 1.59 |
| $P_{m=3,s=2}$ | 2008 | 0.88 | 1.19 | 1.52 | 0.84 | 1.14 | 1.49 |
| $P_{m=3,s=2}$ | 2009 | 1.11 | 1.49 | 1.93 | 1.04 | 1.39 | 1.8 |
| $P_{m=3,s=2}$ | 2010 | 0.94 | 1.25 | 1.63 | 0.93 | 1.31 | 1.79 |
| $P_{m=3,s=2}$ | 2011 | 1.31 | 1.68 | 2.16 | 1.24 | 1.64 | 2.08 |
| $P_{m=3,s=2}$ | 2012 | 1.36 | 1.68 | 2.06 | 1.32 | 1.68 | 2.1 |
| $P_{m=3,s=2}$ | 2013 | 0.89 | 1.15 | 1.46 | 0.86 | 1.11 | 1.43 |
| $P_{m=3,s=2}$ | 2014 | 0.93 | 1.38 | 1.95 | 0.93 | 1.51 | 2.34 |
| $P_{m=3,s=2}$ | 2015 | 0.68 | 0.89 | 1.16 | 0.63 | 0.85 | 1.11 |
| $P_{m=3,s=2}$ | 2016 | 0.83 | 1.24 | 1.79 | 0.73 | 1.11 | 1.63 |
| $P_{m=3,s=2}$ | 2017 | 0.48 | 0.66 | 0.89 | 0.44 | 0.62 | 0.84 |
| $P_{m=3,s=2}$ | 2018 | 1 | 1.27 | 1.56 | 0.92 | 1.17 | 1.47 |
| $P_{m=3,s=2}$ | 2019 | 1.53 | 1.89 | 2.31 | 1.56 | 1.95 | 2.43 |
| $P_{m=3,s=2}$ | 2020 | 1.39 | 1.73 | 2.15 | 1.6 | 2 | 2.46 |
| $P_{m=3,s=2}$ | 2021 | 0.86 | 1.06 | 1.31 | 0.81 | 1.01 | 1.25 |
| $P_{m=3,s=2}$ | 2022 | 0.69 | 0.89 | 1.13 | 0.66 | 0.86 | 1.12 |
| ${P.\mu}_{m=4,s=2}$ | NA | 0.43 | 0.67 | 1.03 | 0.5 | 0.78 | 1.19 |
| $P_{m=4,s=2}$ | 1998 | 0.38 | 0.63 | 0.95 | 0.47 | 0.8 | 1.25 |
| $P_{m=4,s=2}$ | 1999 | 0.19 | 0.29 | 0.41 | 0.23 | 0.36 | 0.56 |
| $P_{m=4,s=2}$ | 2000 | 0.73 | 0.95 | 1.31 | 0.74 | 0.99 | 1.33 |
| $P_{m=4,s=2}$ | 2001 | 1.06 | 1.33 | 1.66 | 1.31 | 1.71 | 2.17 |
| $P_{m=4,s=2}$ | 2002 | 0.25 | 0.36 | 0.49 | 0.26 | 0.4 | 0.59 |
| $P_{m=4,s=2}$ | 2003 | 0.76 | 0.99 | 1.25 | 0.88 | 1.18 | 1.55 |
| $P_{m=4,s=2}$ | 2004 | 0.08 | 0.15 | 0.26 | 0.08 | 0.15 | 0.27 |
| $P_{m=4,s=2}$ | 2005 | 0.76 | 1.14 | 1.46 | 0.69 | 1.06 | 1.5 |
| $P_{m=4,s=2}$ | 2006 | 0.41 | 0.57 | 0.77 | 0.47 | 0.68 | 1 |
| $P_{m=4,s=2}$ | 2007 | 1.1 | 1.56 | 2.09 | 1.07 | 1.56 | 2.14 |
| $P_{m=4,s=2}$ | 2008 | 0.8 | 1.07 | 1.39 | 0.89 | 1.22 | 1.62 |
| $P_{m=4,s=2}$ | 2009 | 0.99 | 1.38 | 1.87 | 1 | 1.41 | 1.96 |
| $P_{m=4,s=2}$ | 2010 | 0.12 | 0.23 | 0.37 | 0.13 | 0.25 | 0.45 |
| $P_{m=4,s=2}$ | 2011 | 1.79 | 2.33 | 2.97 | 2.14 | 2.85 | 3.72 |
| $P_{m=4,s=2}$ | 2012 | 1.04 | 1.36 | 1.73 | 1.19 | 1.61 | 2.13 |
| $P_{m=4,s=2}$ | 2013 | 0.15 | 0.26 | 0.42 | 0.16 | 0.29 | 0.47 |
| $P_{m=4,s=2}$ | 2014 | 0.02 | 0.08 | 0.21 | 0.03 | 0.11 | 0.29 |
| $P_{m=4,s=2}$ | 2015 | 0.46 | 0.66 | 0.93 | 0.48 | 0.7 | 0.97 |
| $P_{m=4,s=2}$ | 2016 | 0.25 | 0.51 | 0.93 | 0.26 | 0.57 | 1.14 |
| $P_{m=4,s=2}$ | 2017 | 0.11 | 0.23 | 0.4 | 0.11 | 0.24 | 0.43 |
| $P_{m=4,s=2}$ | 2018 | 0.08 | 0.16 | 0.29 | 0.09 | 0.18 | 0.32 |
| $P_{m=4,s=2}$ | 2019 | 0.06 | 0.13 | 0.24 | 0.06 | 0.14 | 0.26 |
| $P_{m=4,s=2}$ | 2020 | 0.13 | 0.21 | 0.32 | 0.18 | 0.32 | 0.54 |
| $P_{m=4,s=2}$ | 2021 | 0.11 | 0.19 | 0.3 | 0.12 | 0.22 | 0.38 |
| $P_{m=4,s=2}$ | 2022 | 0.08 | 0.14 | 0.23 | 0.1 | 0.19 | 0.34 |
| ${CS}^{(Act)}$ | 1998 | 12.42 | 15.47 | 17.78 | 11.4 | 14.52 | 17.44 |
| ${CS}^{(Act)}$ | 1999 | 11.7 | 13.78 | 15.95 | 11.4 | 13.52 | 15.69 |
| ${CS}^{(Act)}$ | 2000 | 12.55 | 14.89 | 17.19 | 12.4 | 14.74 | 17.12 |
| ${CS}^{(Act)}$ | 2001 | 11.71 | 14.8 | 17.57 | 11.52 | 14.66 | 17.53 |
| ${CS}^{(Act)}$ | 2002 | 11.06 | 13.76 | 16.67 | 10.89 | 13.6 | 16.62 |
| ${CS}^{(Act)}$ | 2003 | 11.19 | 14.12 | 17.26 | 10.99 | 14 | 17.22 |
| ${CS}^{(Act)}$ | 2004 | 10.32 | 12.01 | 14.55 | 10.27 | 11.83 | 14.47 |
| ${CS}^{(Act)}$ | 2005 | 11.42 | 15.33 | 17.82 | 11.41 | 15.34 | 17.85 |
| ${CS}^{(Act)}$ | 2006 | 10.25 | 11.94 | 15.18 | 10.24 | 11.76 | 15.06 |
| ${CS}^{(Act)}$ | 2007 | 10.35 | 12.35 | 15.45 | 10.35 | 12.49 | 15.72 |
| ${CS}^{(Act)}$ | 2008 | 10.37 | 12.43 | 15.34 | 10.68 | 13.42 | 16.56 |
| ${CS}^{(Act)}$ | 2009 | 12.48 | 14.63 | 16.84 | 12.67 | 14.89 | 17.13 |
| ${CS}^{(Act)}$ | 2010 | 10.48 | 12.72 | 15.55 | 10.57 | 12.93 | 15.89 |
| ${CS}^{(Act)}$ | 2011 | 11.09 | 12.6 | 14.11 | 10.88 | 12.42 | 14.01 |
| ${CS}^{(Act)}$ | 2012 | 12.74 | 14.97 | 17.27 | 12.49 | 14.76 | 17.07 |
| ${CS}^{(Act)}$ | 2013 | 11.18 | 13.23 | 15.39 | 11.45 | 13.59 | 15.79 |
| ${CS}^{(Act)}$ | 2014 | 10.31 | 11.9 | 14.17 | 10.3 | 11.85 | 14.16 |
| ${CS}^{(Act)}$ | 2015 | 11.54 | 12.75 | 13.98 | 11.55 | 12.75 | 13.97 |
| ${CS}^{(Act)}$ | 2016 | 10.39 | 12.6 | 15.69 | 10.7 | 13.41 | 16.52 |
| ${CS}^{(Act)}$ | 2017 | 10.42 | 12.64 | 15.71 | 10.75 | 13.54 | 16.93 |
| ${CS}^{(Act)}$ | 2018 | 10.27 | 12.05 | 15.37 | 10.29 | 12.28 | 15.96 |
| ${CS}^{(Act)}$ | 2019 | 10.82 | 13.46 | 16.56 | 11.1 | 13.99 | 17.07 |
| ${CS}^{(Act)}$ | 2020 | 11.66 | 13.75 | 15.9 | 11.83 | 13.88 | 16.04 |
| ${CS}^{(Act)}$ | 2021 | 12.21 | 14.14 | 16.19 | 12.98 | 14.85 | 16.83 |
| ${CS}^{(Act)}$ | 2022 | 11.76 | 12.28 | 12.81 | 11.87 | 12.4 | 12.92 |
| ${Availability}^{(Act)}$ | 1998 | 0.72 | 0.81 | 0.88 | 0.74 | 0.82 | 0.89 |
| ${Availability}^{(Act)}$ | 1999 | 0.62 | 0.81 | 0.93 | 0.66 | 0.83 | 0.94 |
| ${Availability}^{(Act)}$ | 2000 | 0.64 | 0.83 | 0.94 | 0.66 | 0.84 | 0.94 |
| ${Availability}^{(Act)}$ | 2001 | 0.77 | 0.91 | 0.97 | 0.77 | 0.91 | 0.97 |
| ${Availability}^{(Act)}$ | 2002 | 0.78 | 0.92 | 0.98 | 0.79 | 0.92 | 0.98 |
| ${Availability}^{(Act)}$ | 2003 | 0.78 | 0.93 | 0.98 | 0.79 | 0.93 | 0.98 |
| ${Availability}^{(Act)}$ | 2004 | 0.84 | 0.95 | 0.99 | 0.85 | 0.95 | 0.99 |
| ${Availability}^{(Act)}$ | 2005 | 0.61 | 0.78 | 0.91 | 0.61 | 0.78 | 0.91 |
| ${Availability}^{(Act)}$ | 2006 | 0.74 | 0.88 | 0.96 | 0.75 | 0.89 | 0.96 |
| ${Availability}^{(Act)}$ | 2007 | 0.77 | 0.92 | 0.98 | 0.76 | 0.92 | 0.98 |
| ${Availability}^{(Act)}$ | 2008 | 0.83 | 0.95 | 0.99 | 0.8 | 0.94 | 0.99 |
| ${Availability}^{(Act)}$ | 2009 | 0.83 | 0.95 | 0.99 | 0.8 | 0.94 | 0.99 |
| ${Availability}^{(Act)}$ | 2010 | 0.77 | 0.91 | 0.97 | 0.76 | 0.91 | 0.97 |
| ${Availability}^{(Act)}$ | 2011 | 0.77 | 0.84 | 0.9 | 0.78 | 0.85 | 0.9 |
| ${Availability}^{(Act)}$ | 2012 | 0.86 | 0.93 | 0.97 | 0.86 | 0.94 | 0.97 |
| ${Availability}^{(Act)}$ | 2013 | 0.92 | 0.98 | 1 | 0.91 | 0.98 | 1 |
| ${Availability}^{(Act)}$ | 2014 | 0.79 | 0.92 | 0.98 | 0.79 | 0.92 | 0.98 |
| ${Availability}^{(Act)}$ | 2015 | 0.76 | 0.95 | 0.99 | 0.76 | 0.94 | 0.99 |
| ${Availability}^{(Act)}$ | 2016 | 0.85 | 0.97 | 1 | 0.85 | 0.97 | 1 |
| ${Availability}^{(Act)}$ | 2017 | 0.83 | 0.96 | 0.99 | 0.8 | 0.96 | 0.99 |
| ${Availability}^{(Act)}$ | 2018 | 0.87 | 0.97 | 1 | 0.86 | 0.97 | 1 |
| ${Availability}^{(Act)}$ | 2019 | 0.87 | 0.97 | 0.99 | 0.87 | 0.97 | 0.99 |
| ${Availability}^{(Act)}$ | 2020 | 0.91 | 0.97 | 0.99 | 0.91 | 0.97 | 0.99 |
| ${Availability}^{(Act)}$ | 2021 | 0.92 | 0.99 | 1 | 0.91 | 0.98 | 1 |
| ${Availability}^{(Act)}$ | 2022 | 0.93 | 0.98 | 1 | 0.88 | 0.98 | 1 |
| ${p.det}^{(Act)}$ | NA | 0.99 | 0.99 | 1 | 0.99 | 0.99 | 1 |
| $\gamma^{(B)}$ | NA | -5.E-04 | -1.E-04 | -4.E-06 | -4.E-04 | -1.E-04 | -1.E-05 |
| $\gamma^{(NB)}$ | NA | -2.E-04 | -4.E-05 | -2.E-06 | -1.E-04 | -3.E-05 | -1.E-06 |
| $\gamma^{(prod)}$ | NA | -1.E-03 | -7.E-04 | -4.E-04 | -1.E-03 | -9.E-04 | -4.E-04 |
