# Supplementary material for: Use of integrated population models for assessing density-dependence and juvenile survival in Northern Bobwhites (Colinus virginianus)
Source: PeerJ. 2024 Dec 4;12:e18625. doi: 10.7717/peerj.18625 (PMC11624843; doi:10.7717/peerj.18625)
Supplement: Supplemental Information 2 — Posterior samples were generated from an Integrated Population Model using demographic data from a bobwhite population in southern Georgia, USA, 1998–2022. Years were classified as positive (2001, 2012, 2015, 2019) or negative (1999, 2004, 2006, 2014) if the 95% credible intervals of the estimated April population growth rate ( <!--[if !msEquation]--> <!--[endif]-->) were greater or lesser than 1, respectively. Estimates are show for: April population density (birds/ha, <!--[if !msEquation]--> <!--[endif]-->), bi-weekly breeding survival ( <!--[if !msEquation]--> <!--[endif]-->), bi-weekly non-breeding survival ( <!--[if !msEquation]--> <!--[endif]-->), daily juvenile survival ( <!--[if !msEquation]--> <!--[endif]-->), monthly per-capita productivity ( <!--[if !msEquation]--> <!--[endif]-->), and total per-capita productivity ( <!--[if !msEquation]--> <!--[endif]-->). Subscripts for vital rates correspond to month of the breeding season (m, 1 = June, 2 = July, 3 = August, 4 = September), age during the non-breeding season (a, 1 = adult, 2 = subadult), and sex (s, 1 = male, 2 = female). Parameters that differ between years of increasing and decreasing population growth are bolded. [file peerj-12-18625-s002.docx]

|  | Increasing | | | Decreasing | | |
| --- | --- | --- | --- | --- | --- | --- |
| Parameter | Mean | Lower | Upper | Mean | Lower | Upper |
| $\boldsymbol{D}^{\boldsymbol{(Apr)}}$ | **3.06** | **2.95** | **3.41** | **3.59** | **3.46** | **3.96** |
| $\boldsymbol{\phi}^{\boldsymbol{(B)}}$ | **0.89** | **0.885** | **0.901** | **0.852** | **0.848** | **0.864** |
| $\phi_{a=1,s=1}^{\left( NB \right)}$ | 0.927 | 0.923 | 0.94 | 0.927 | 0.923 | 0.939 |
| $\boldsymbol{\phi}_{\boldsymbol{a=1,s=2}}^{\left( \boldsymbol{NB} \right)}$ | **0.933** | **0.926** | **0.951** | **0.895** | **0.887** | **0.919** |
| $\phi_{a=2,s=1}^{\left( NB \right)}$ | 0.921 | 0.916 | 0.935 | 0.905 | 0.898 | 0.922 |
| $\boldsymbol{\phi}_{\boldsymbol{a=2,s=2}}^{\left( \boldsymbol{NB} \right)}$ | **0.949** | **0.946** | **0.96** | **0.896** | **0.889** | **0.916** |
| $\phi_{m=1}^{\left( J.daily \right)}$ | 0.992 | 0.992 | 0.993 | 0.991 | 0.99 | 0.992 |
| $\phi_{m=2}^{\left( J.daily \right)}$ | 0.993 | 0.993 | 0.994 | 0.993 | 0.993 | 0.994 |
| $\phi_{m=3}^{\left( J.daily \right)}$ | 0.99 | 0.989 | 0.991 | 0.99 | 0.989 | 0.992 |
| $\phi_{m=4}^{\left( J.daily \right)}$ | 0.992 | 0.992 | 0.994 | 0.993 | 0.992 | 0.994 |
| $P_{m=1,s=1}$ | 0.67 | 0.57 | 1.04 | 0.96 | 0.89 | 1.17 |
| $\boldsymbol{P}_{\boldsymbol{m=1,s=2}}$ | **2.93** | **2.85** | **3.15** | **1.97** | **1.92** | **2.13** |
| $P_{m=2,s=1}$ | 0.35 | 0.29 | 0.54 | 0.38 | 0.31 | 0.61 |
| $P_{m=2,s=2}$ | 1.56 | 1.51 | 1.71 | 1.59 | 1.53 | 1.76 |
| $\boldsymbol{P}_{\boldsymbol{m=3,s=1}}$ | **0.35** | **0.31** | **0.48** | **0.05** | **0.02** | **0.16** |
| $\boldsymbol{P}_{\boldsymbol{m=3,s=2}}$ | **1.73** | **1.67** | **1.91** | **1.25** | **1.19** | **1.44** |
| $P_{m=4,s=1}$ | 0.02 | 0.004 | 0.05 | 0.01 | 0.004 | 0.04 |
| $\boldsymbol{P}_{\boldsymbol{m=4,s=2}}$ | **0.88** | **0.83** | **1.01** | **0.28** | **0.26** | **0.34** |
| $P_{s=1}^{\left( Tot \right)}$ | 1.03 | 0.92 | 1.36 | 0.99 | 0.92 | 1.2 |
| $\boldsymbol{P}_{\boldsymbol{s=2}}^{\left( \boldsymbol{Tot} \right)}$ | **4.98** | **4.86** | **5.35** | **3.27** | **3.19** | **3.51** |
