# Supplementary material for: Use of integrated population models for assessing density-dependence and juvenile survival in Northern Bobwhites (Colinus virginianus)
Source: PeerJ. 2024 Dec 4;12:e18625. doi: 10.7717/peerj.18625 (PMC11624843; doi:10.7717/peerj.18625)
Supplement: Supplemental Information 3 [file peerj-12-18625-s003.docx]

The model requires accurate estimates of chick production; however, 17% of successful nests did not have information recorded about the number of chicks hatched. We performed a preliminary analysis to estimate the number of chicks hatched from nests with missing data. The number of chicks successfully hatched from nest $n$ (${Hatch}_{n}$) was dependent on the clutch size (${Clutch}_{n}$) and the mean hatching rate ($p_{m,t}^{\left( hatch \right)}$), which we estimated for each month and year:

$${Hatch}_{n}\sim Binomial\left( {Clutch}_{n},p_{m,t}^{\left( hatch \right)} \right)$$

We then used the estimates of $p_{m,t}^{\left( hatch \right)}$ to predict ${Hatch}_{n}$ for nests with missing data. About 45% of the successful nests with missing hatch data were also missing data on clutch size. In these cases, we predicted the missing clutch size similar to above with a Poisson distribution:

$${Clutch}_{n}\sim Poisson\left( \lambda_{m,t}^{\left( clutch \right)} \right)$$

We used only successful nests to inform $p_{m,t}^{\left( hatch \right)}$, but used all nests (successful and failed) with clutch size information to inform $\lambda_{m,t}^{\left( clutch \right)}$. We assumed that clutch sizes and hatch rates would be similar across sexes. We then summed the observed and predicted number of chicks across sexes, months, and years, and calculated the mean ($\mu_{m,s,t}^{\left( Chicks.est \right)}$) and sd ($\sigma_{m,s,t}^{\left( Chicks.est \right)}$) of the posterior samples.
