# Supplementary material for: Use of integrated population models for assessing density-dependence and juvenile survival in Northern Bobwhites (Colinus virginianus)
Source: PeerJ. 2024 Dec 4;12:e18625. doi: 10.7717/peerj.18625 (PMC11624843; doi:10.7717/peerj.18625)
Supplement: Supplemental Information 4 [file peerj-12-18625-s004.docx]

The detection process can be decomposed into two processes (Amundson et al. 2014). The first is availability (${Availability}_{t}^{\left( Act \right)}$), which is the probability of a bobwhite covey calling during the survey and thus being able to be detected. Second, detection (${p.det}^{(Act)}$) is the probability of observers hearing and recording a covey, conditional on it being available. Calling rates of bobwhites coveys are positively related to covey density (Wellendorf et al. 2004). We calculated availability for each grid cell in each year based on the observed neighbor density on surveys (coveys detected – 1) and the logistic regression calling rate parameters reported by Wellendorf et al. (2004). Mean availability varied between 0.52 – 1 across grids and years. In contrast to availability, we assumed that conditional detection probability would not vary over space and time. We used data and model code provided in Howell et al. (2021) to estimate the mean and standard deviation of the probability of at least one of the four observers detecting an available covey. The detection radius for calling bobwhites is close to the width of the grid cells (Sisson et al. 2017, Howell et al. 2021), so the probability of at least one observer detecting a calling bobwhite was near 1 (mean: 0.99, sd: 0.001). We incorporated theses parameters as informative priors on the true detection probability (${p.det}^{(Act)}$) and yearly availability (${Availability}_{t}^{\left( Act \right)}$).
