# Supplementary material for: Use of integrated population models for assessing density-dependence and juvenile survival in Northern Bobwhites (Colinus virginianus)
Source: PeerJ. 2024 Dec 4;12:e18625. doi: 10.7717/peerj.18625 (PMC11624843; doi:10.7717/peerj.18625)
Supplement: Supplemental Information 5 [file peerj-12-18625-s005.docx]

We implemented two varieties of the transient life-table response experiments of (Koons et al. 2016, 2017), corresponding to the random-effect and fixed-effect designs of Caswell (2001). The random-design approach decomposes the variance of the realized growth rate due to the variance and covariance of the demographic rates and population age and sex structure. This method was implemented by calculating sensitivities of population growth to the parameters, assessed as the first derivative of the realized population growth rate based on the mean values of each vital rate and proportional age and sex structure of the population in April ($S_{a,s,t}/sum(S_{1:2,1:2,t}$). The contributions of each parameter to population growth were then calculated as the sum of the products of the sensitivities and the variance and covariance between parameters. The fixed-design approach assessed how the change in realized growth rate between successive years were driven by changes in demographic rates and population structure. The sensitivities were calculated as above, though based on the mean of parameters across successive years rather than across the entire study duration. Demographic contributions were then calculated as the product of the sensitivities and the differences between parameters across successive years. Both tLTRE variants were implemented based on code in Kéry and Schaub (2021).
