# Supplementary figures and images for: Use of integrated population models for assessing density-dependence and juvenile survival in Northern Bobwhites (Colinus virginianus)
Source: PeerJ. 2024 Dec 4;12:e18625. doi: 10.7717/peerj.18625 (PMC11624843; doi:10.7717/peerj.18625)

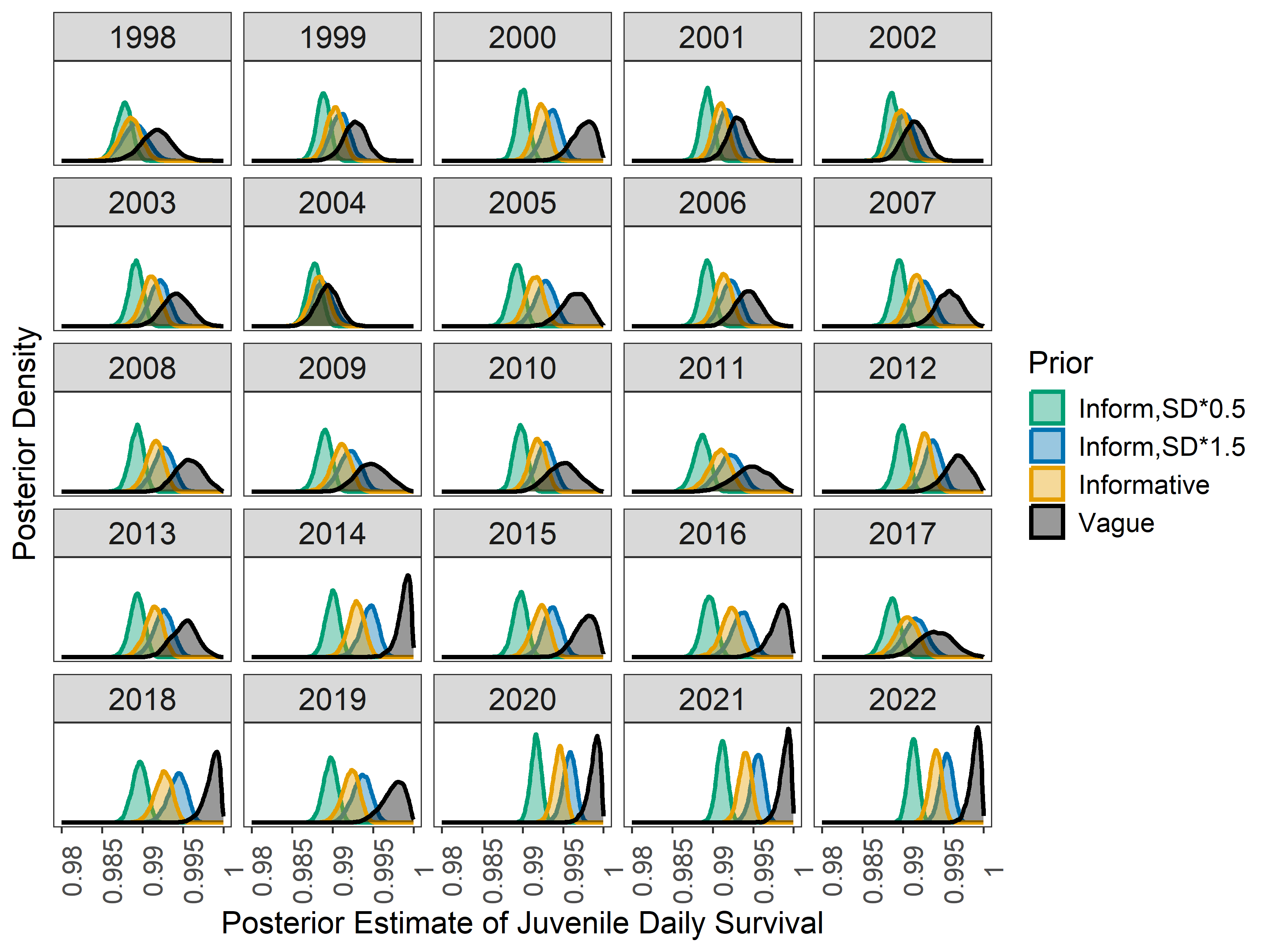

Supplement: Supplemental Information 6 — Results show the effect of prior specification on the posterior distribution for the juvenile survival parameter estimated via an integrated population model. The prior distribution was specified as either fully vague (black), using on an informative mean and standard deviation derived from Terhune, Chandler & Martin, 2017 (orange), and using the same informative mean but varying the standard deviation up (blue) or down (green) by 50%. [file peerj-12-18625-s006.png]

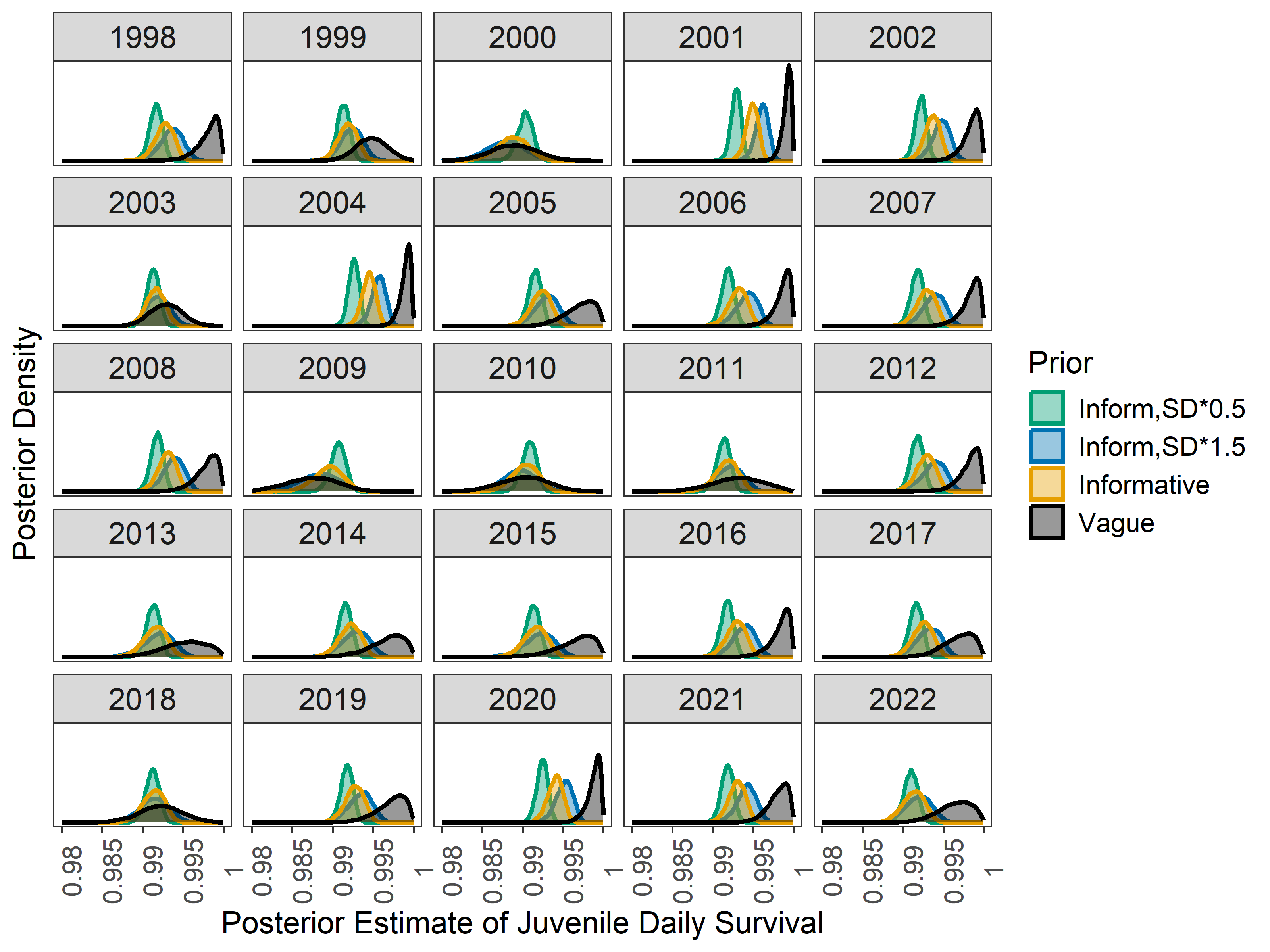

Supplement: Supplemental Information 7 — Results show the effect of prior specification on the posterior distribution for the juvenile survival parameter estimated via an integrated population model. The prior distribution was specified as either fully vague (black), using on an informative mean and standard deviation derived from Terhune, Chandler & Martin, 2017 (orange), and using the same informative mean but varying the standard deviation up (blue) or down (green) by 50%. [file peerj-12-18625-s007.png]

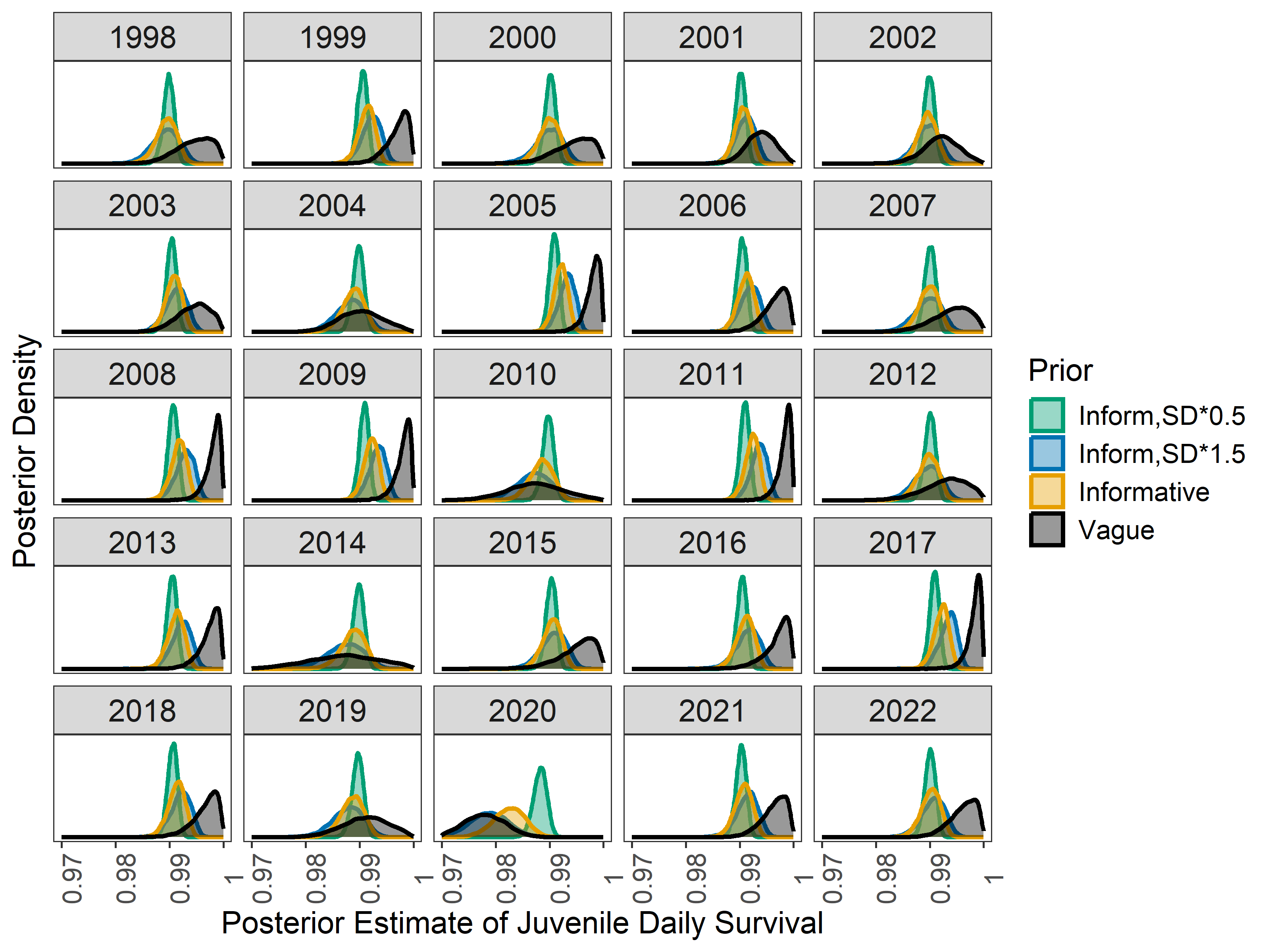

Supplement: Supplemental Information 8 — Results show the effect of prior specification on the posterior distribution for the juvenile survival parameter estimated via an integrated population model. The prior distribution was specified as either fully vague (black), using on an informative mean and standard deviation derived from Terhune, Chandler & Martin, 2017 (orange), and using the same informative mean but varying the standard deviation up (blue) or down (green) by 50%. [file peerj-12-18625-s008.png]

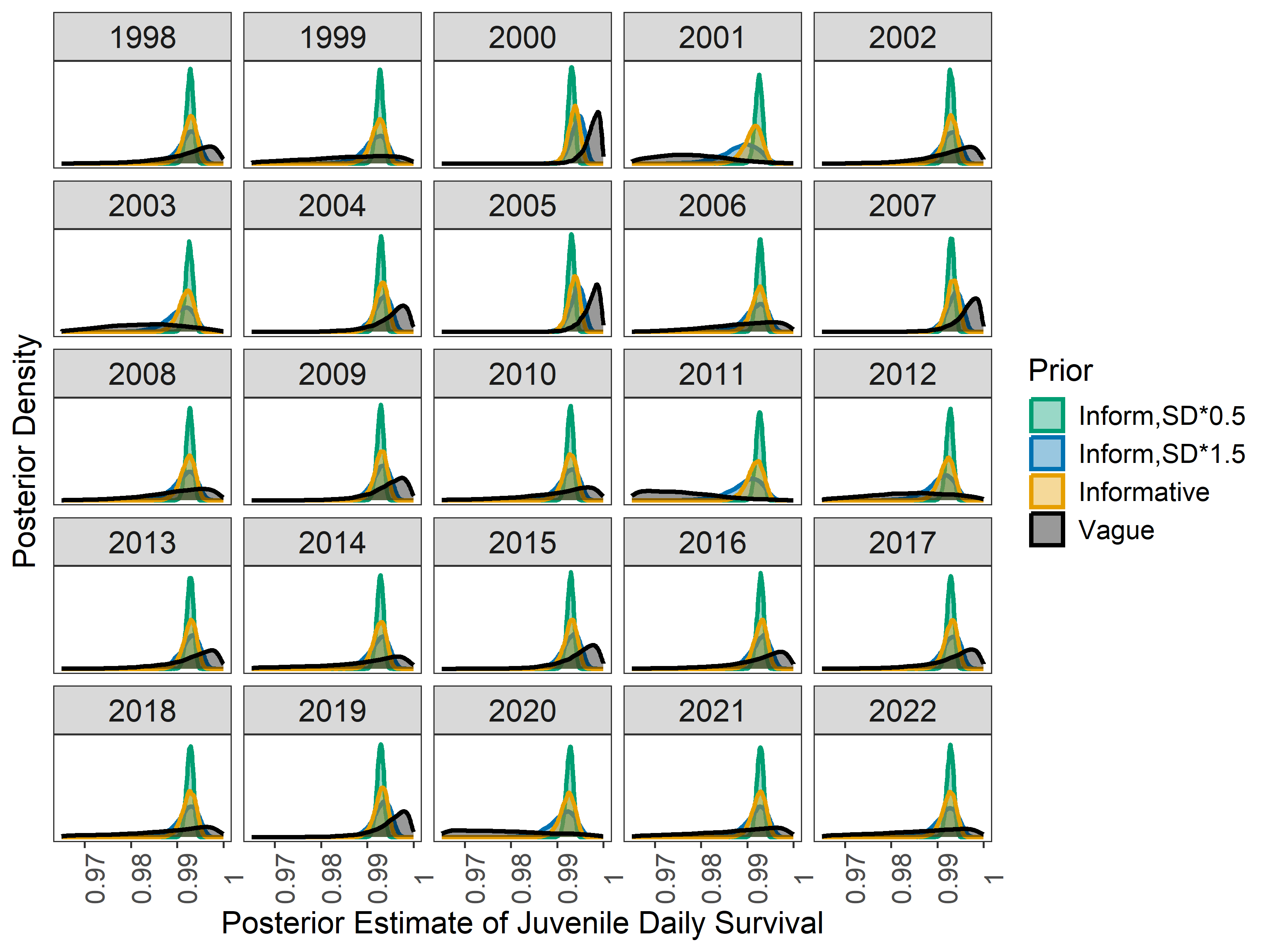

Supplement: Supplemental Information 9 — Results show the effect of prior specification on the posterior distribution for the juvenile survival parameter estimated via an integrated population model. The prior distribution was specified as either fully vague (black), using on an informative mean and standard deviation derived from Terhune, Chandler & Martin, 2017 (orange), and using the same informative mean but varying the standard deviation up (blue) or down (green) by 50%. [file peerj-12-18625-s009.png]

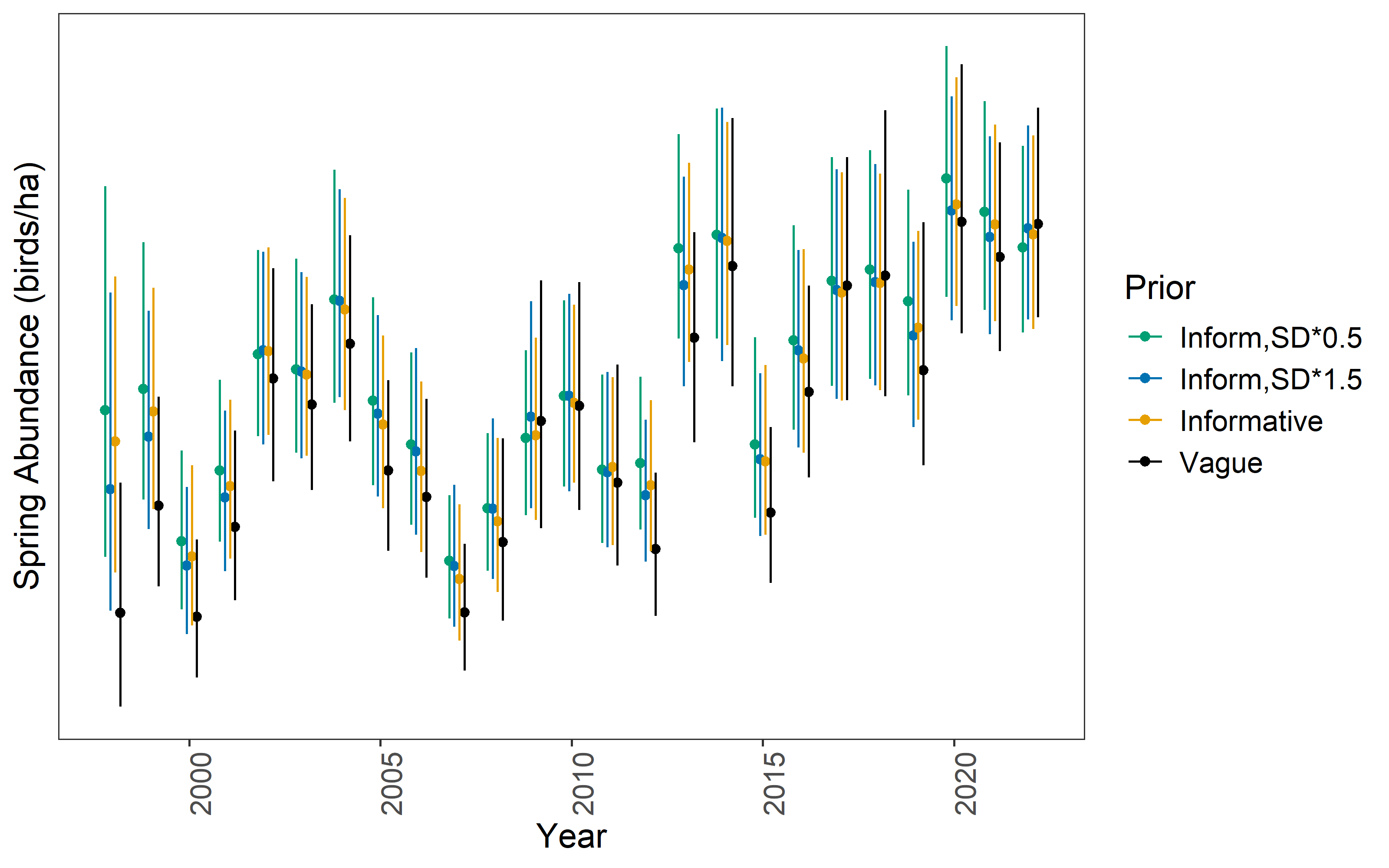

Supplement: Supplemental Information 10 — The prior distribution was specified as either fully vague (black), using on an informative mean and standard deviation derived from Terhune, Chandler & Martin, 2017 (orange), and using the same informative mean but varying the standard deviation up (blue) or down (green) by 50% in an integrated population model. [file peerj-12-18625-s010.png]

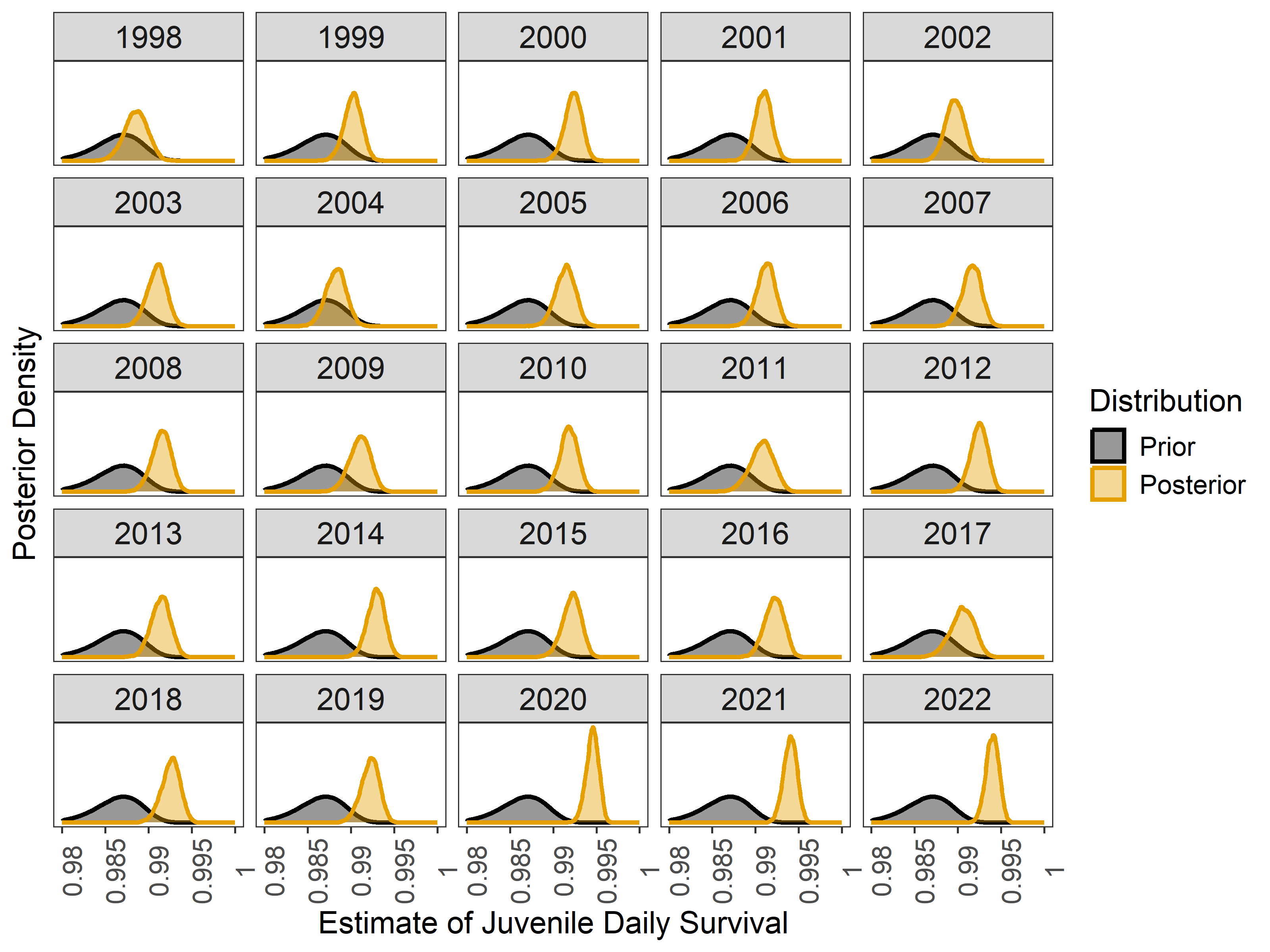

Supplement: Supplemental Information 11 — The prior distribution was specified using an informative mean and standard deviation derived from Terhune, Chandler & Martin, 2017. [file peerj-12-18625-s011.png]

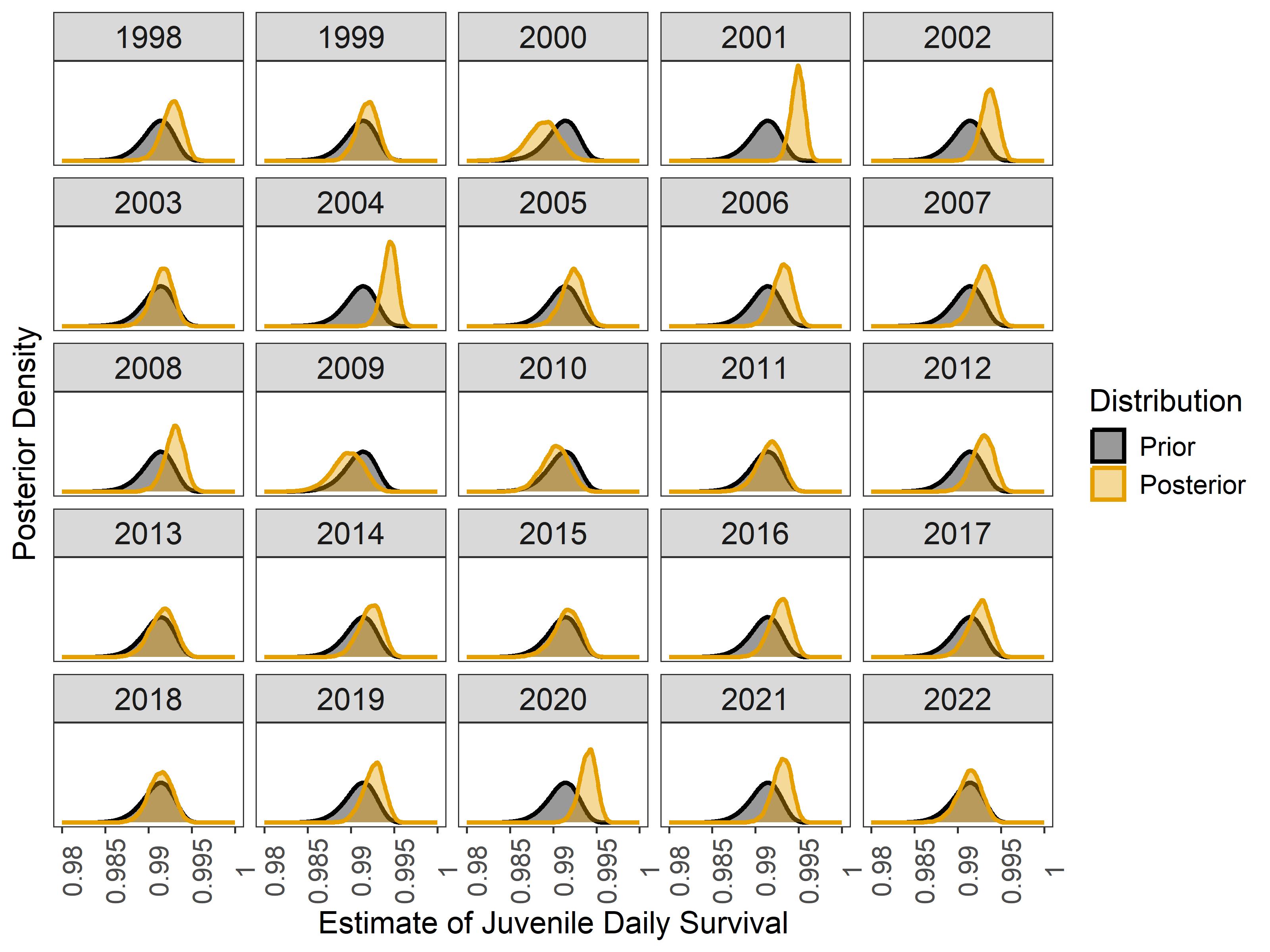

Supplement: Supplemental Information 12 — The prior distribution was specified using an informative mean and standard deviation derived from Terhune, Chandler & Martin, 2017. [file peerj-12-18625-s012.png]

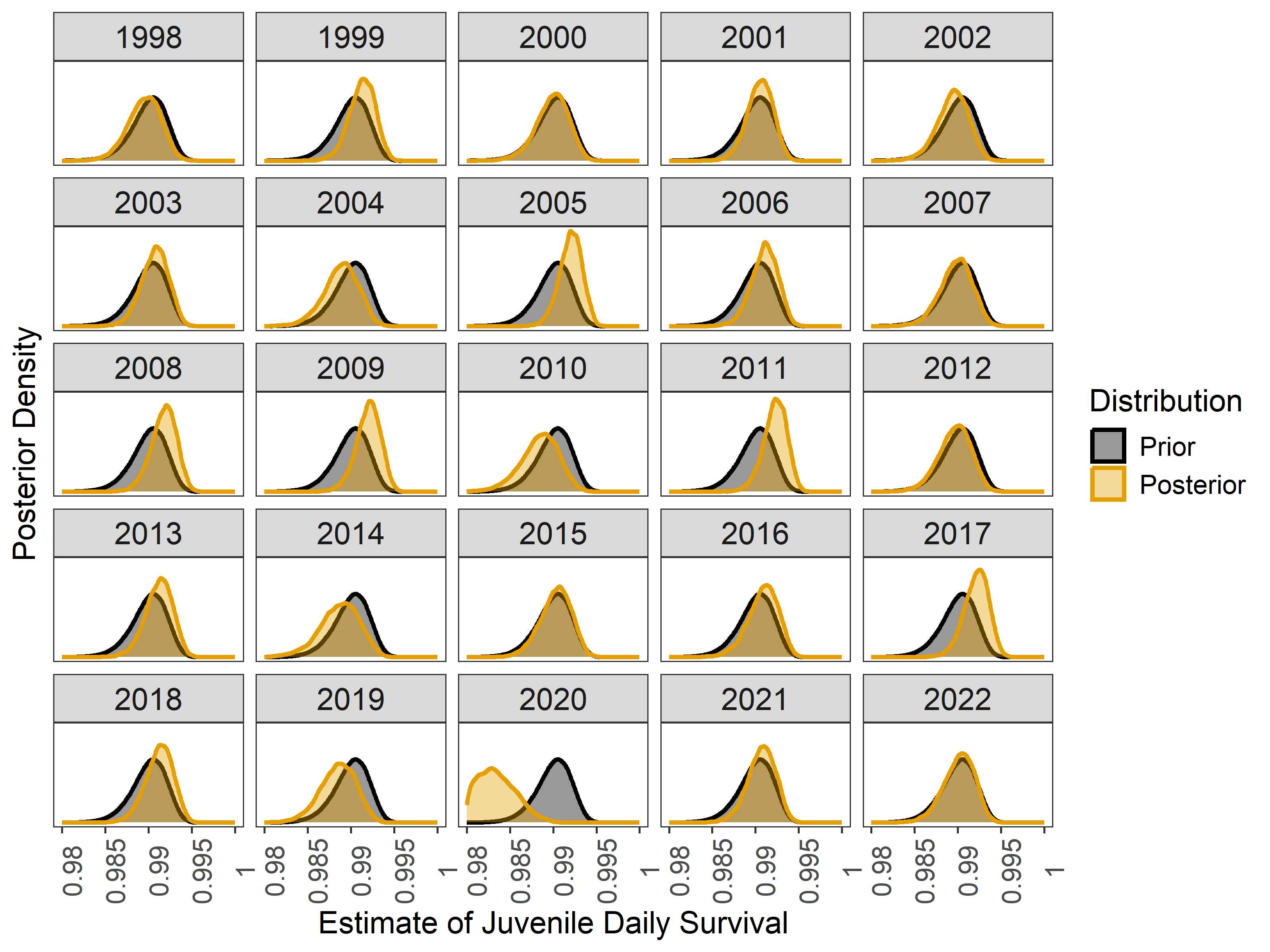

Supplement: Supplemental Information 13 — The prior distribution was specified using an informative mean and standard deviation derived from Terhune, Chandler & Martin, 2017. [file peerj-12-18625-s013.png]

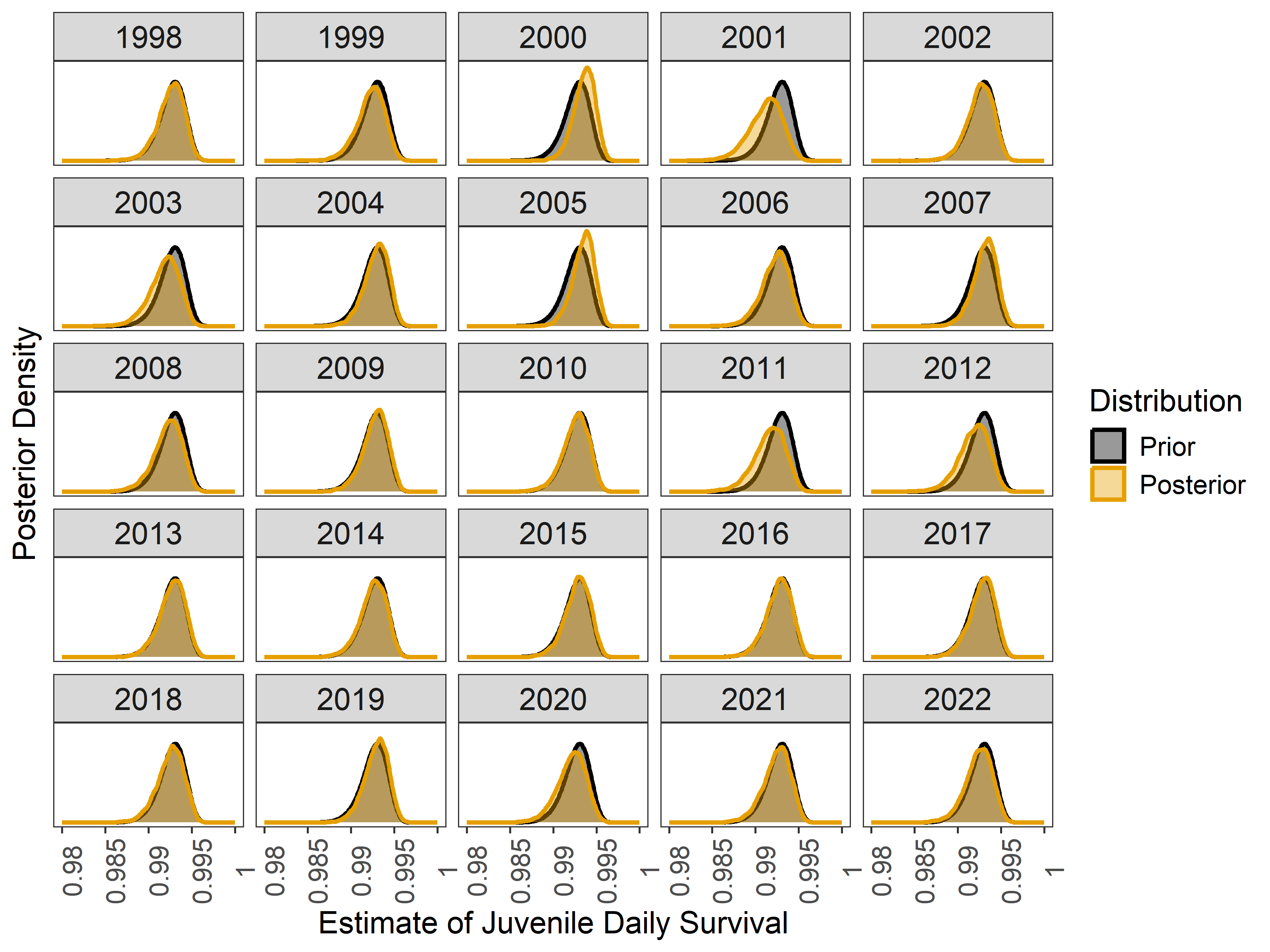

Supplement: Supplemental Information 14 — The prior distribution was specified using an informative mean and standard deviation derived from Terhune, Chandler & Martin, 2017. [file peerj-12-18625-s014.png]

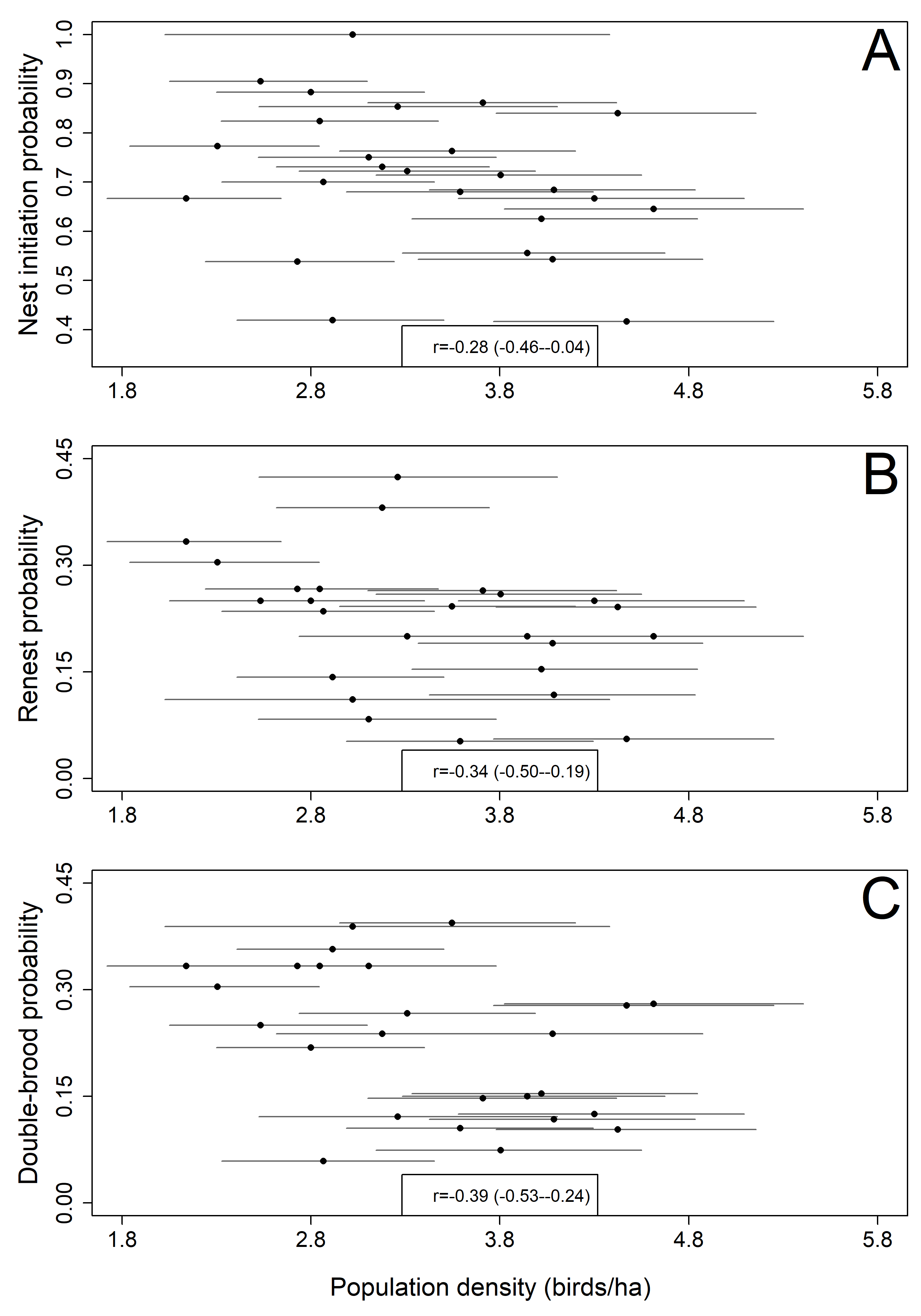

Supplement: Supplemental Information 15 — Correlations were generated between posterior samples of April density derived from an integrated population model and the observed percentage of radiotracked females surviving the breeding season which initiated nests (A), the observed percentage of nesting females surviving the breeding season which initiated renest attempts after a failed first brood (B), or the observed percentage of nesting females surviving the breeding season which initiated double-brood attempts after a successful first brood (C). Points represent the mean of posterior samples for April density (x-axis) and the mean of observed reproduction parameters (y-axis). Horizontal bars represent the 95% credible intervals of posterior samples for April density. Mean correlation coefficients are shown, as well as 95% credible intervals in parentheses. [file peerj-12-18625-s015.png]

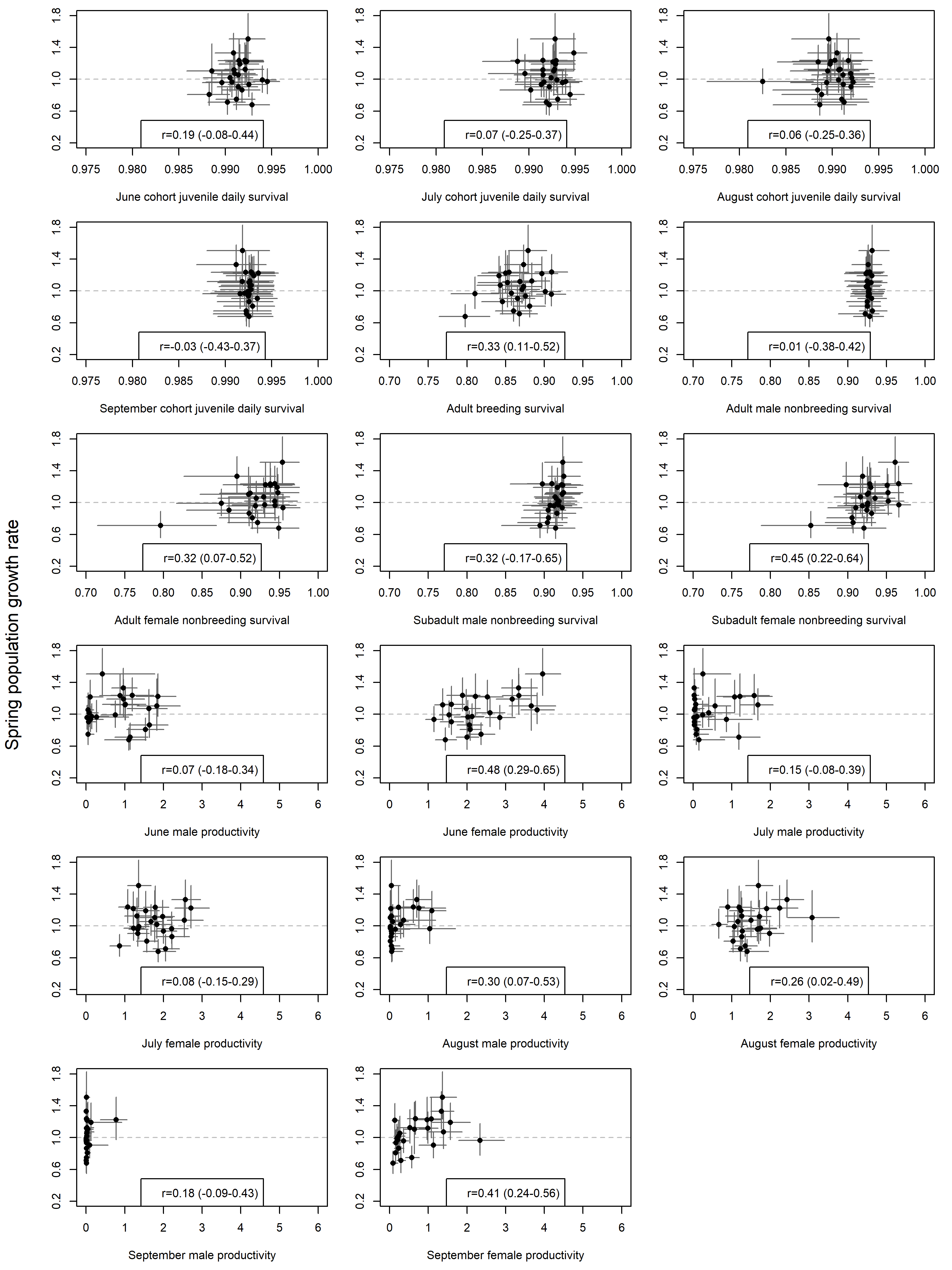

Supplement: Supplemental Information 16 — Correlations were generated between posterior samples derived from an integrated population model. Points represent the mean of posterior samples for population growth rates and demographic parameters. Horizontal and vertical bars represent the 95% credible intervals of posterior samples for demographic rates and population growth rates, respectively. Mean correlation coefficients are shown, as well as 95% credible intervals in parentheses. Dashed horizontal lines represent no population growth (lambda=0). [file peerj-12-18625-s016.png]
